# Comparison of historic and novel data reveals higher contemporary diversity of trematode metacercariae in freshwater fish

**DOI:** 10.1051/parasite/2025067

**Published:** 2026-01-06

**Authors:** Olena Kudlai, Rasa Binkienė, Vytautas Rakauskas, Nathan Jay Baker

**Affiliations:** State Scientific Research Institute Nature Research Centre Vilnius 08412 Lithuania

**Keywords:** Trematoda, Diversity, Intermediate hosts, Mitochondrial and nuclear DNA, Lithuania

## Abstract

Trematode metacercariae are the most abundant and frequently encountered helminths in freshwater fish. Yet, accurate species identification remains challenging, potentially leading to an underestimation of trematode diversity. Using data from parasitological examinations of 1,030 fish (47 species) collected from diverse freshwater habitats in Lithuania (2022–2024), we assessed the contemporary diversity of trematode metacercariae, host associations, microhabitat preferences, and changes in diversity patterns and transmission dynamics. Through integrated morphological and molecular techniques, we identified metacercariae belonging to 51 species from eight families, more than doubling previously reported diversity (25 species). While trematode family composition remained largely unchanged – the Diplostomidae and Strigeidae remained the most diverse families – notable differences were observed at the species level. Metacercariae of the Echinochasmidae and Echinostomatidae were detected for the first time, while previously reported Clinostomidae were absent. Fish of the Leuciscidae hosted the highest trematode diversity. Host specificity of metacercariae was generally low, with most species being euryxenous. At the microhabitat level, eyes harboured the highest number of species, while muscles showed the highest metacercarial density. Notably, we detected species first genetically characterised in North America (*Echinoparyphium* sp. 2 and *Ichthyocotylurus* sp. 2) and species potentially belonging to the genus *Neogogatea*, previously known only from Asia and North America, highlighting potential invasion risks and suggesting that European trematode diversity remains substantially underestimated. Future efforts should obtain molecular data from correctly identified adult specimens to resolve the identity of species currently identified only to the genus or family level, thereby enabling assessment of their geographical distributions and ecological roles.

## Abstract (fr)

Les métacercaires de Trématodes sont les helminthes les plus abondants et les plus fréquemment rencontrés chez les poissons d’eau douce. Cependant, l’identification précise des espèces reste difficile, ce qui peut conduire à une sous-estimation de la diversité des Trématodes. À partir des données issues d’examens parasitologiques de 1 030 poissons (47 espèces) prélevés dans divers habitats d’eau douce en Lituanie (2022-2024), nous avons évalué la diversité contemporaine des métacercaires de Trématodes, les associations d’hôtes, les préférences de microhabitat et l’évolution des profils de diversité et de la dynamique de transmission. Grâce à des techniques morphologiques et moléculaires intégrées, nous avons identifié des métacercaires appartenant à 51 espèces dans huit familles, soit plus du double de la diversité précédemment rapportée (25 espèces). Si la composition des familles de trématodes est restée largement inchangée, les Diplostomidae et les Strigeidae restant les familles les plus diversifiées, des différences notables ont été observées au niveau des espèces. Des métacercaires d’Echinochasmidae et d’Echinostomatidae ont été détectées pour la première fois, tandis que les Clinostomidae précédemment signalés étaient absents. Les poissons Leuciscidae abritaient la plus grande diversité de Trématodes. La spécificité d’hôte des métacercaires était généralement faible, la plupart des espèces étant euryxènes. Au niveau du microhabitat, les yeux abritaient le plus grand nombre d’espèces, tandis que les muscles présentaient la plus forte densité de métacercaires. Nous avons notamment détecté des espèces caractérisées génétiquement pour la première fois en Amérique du Nord (*Echinoparyphium* sp. 2 et *Ichthyocotylurus* sp. 2) et des espèces appartenant potentiellement au genre *Neogogatea*, jusqu’alors connues uniquement d’Asie et d’Amérique du Nord, soulignant ainsi les risques potentiels d’invasion et suggérant que la diversité des trématodes européens reste largement sous-estimée. Les efforts futurs devraient permettre d’obtenir des données moléculaires à partir de spécimens adultes correctement identifiés afin de déterminer l’identité des espèces actuellement identifiées uniquement au niveau du genre ou de la famille, permettant ainsi d’évaluer leur répartition géographique et leurs rôles écologiques.

## Introduction

Trematode larvae, metacercariae, are common parasites in freshwater fish [24, 78]. Metacercariae represent an intermediate phase between the dispersive cercarial larvae and adults [22], being considered a “resting” stage because their ability to infect definitive hosts is retained and extends over a relatively long period of time [58]. Metacercariae can be found in or on nearly every fish organ. They tend to show microhabitat preferences (*i.e.* site in host) within their fish host [11, 12, 29, 32, 50, 93], which may overlap with that of other parasite species. These preferences may depend on resource availability, intra- and interspecific competition, host immune response, and microhabitat conditions such as temperature and pH [9]. Furthermore, the presence of metacercariae at certain sites of infection may influence the behaviour of their host and predispose them to predation [45], thereby increasing the likelihood of infection of the final host. The pathogenicity of metacercariae also greatly depends on their density within a given site of infection. Infection with metacercariae can affect fish health and growth, make fish more vulnerable to other infections, and even result in mass fish mortality [35, 46, 49, 60].

As part of the complex life cycles of trematodes, metacercariae serve as ecological indicators, reflecting the presence of host taxa in their ecosystems (*e.g.* molluscs, fish, birds, and mammals). They can also be used to investigate trematode diversity when adults are not available for study and they often alter predator-prey interactions, making them important structuring forces in ecological food webs [20, 47]. Furthermore, monitoring species diversity of metacercariae in freshwater fish can be used to evaluate ecological changes over time, an increasingly important metric used to track the effects of human impacts on biodiversity [55, 86]. Lastly, knowledge on trematode diversity is important to identify any trematode species co-introduced to local ecosystems with their non-native hosts [44, 70]. Therefore, trematodes, including their larval stages, have become increasingly recognised as important indicators for environmental changes.

Ecological, epidemiological, and conservation research are all heavily reliant upon precise species identification. However, the primary challenge when working with metacercariae from freshwater fish is that accurate species identification is difficult. Although metacercariae differ in shape and size, they do not resemble their adult stages and offer few morphological features that could be used for their precise species identification. Furthermore, there is a dearth of information (*e.g.*, detailed identification keys or reference specimens) on metacercariae compared to cercarial or adult trematode stages. Thus, it is tempting for non-specialists to identify species of metacercariae based on their fish host identity or microhabitat, thereby often leading to their misidentification. Surrendering to the inability to reliably identify metacercariae, researchers often identify metacercariae to the order, family, or genus level. Consequently, the current diversity of trematodes could be underestimated.

However, with the advent of molecular methods, together with an ever-growing trematode database in GenBank, searching for a corresponding DNA sequence of metacercariae has become one of the most essential tools for the identification of these trematode larval stages. Presently, however, the GenBank database of trematodes is far from complete, with submissions of sequences generated from accurately identified adults being an imperative prerequisite.

Integrating morphological and molecular techniques, the present study aimed to (i) comprehensively assess the diversity of metacercariae in freshwater fish from Lithuania, a region where contemporary fish parasitological research has rarely been performed, and (ii) compare the newly obtained data to the existing literature on fish parasites from Lithuania to identify and document changes in the diversity of trematode metacercariae and their host associations and transmission pathways. To assess the taxonomic identity of metacercariae, DNA sequences were obtained from as many metacercarial morphotypes from as many host species (and microhabitats within these hosts) collected in as many different localities as possible in various types of freshwater ecosystems. We strived to (i) document which fish hosts are exploited by which metacercariae, including identifying their precise microhabitat within their fish hosts, and (ii) provide vouchers (photogenophores) for the majority of molecularly identified metacercariae. This study provides a novel and comprehensive overview of metacercariae of freshwater fishes from an underrepresented region of Europe, with the data generated herein poised to guide future assessments of metacercariae in freshwater fish, particularly in studies that focus on answering broader ecological, epidemiological, and conservation questions, as well as for studies focused on elucidating trematode life cycles.

## Materials and methods

### Study area and fish sampling

**Figure 1:**
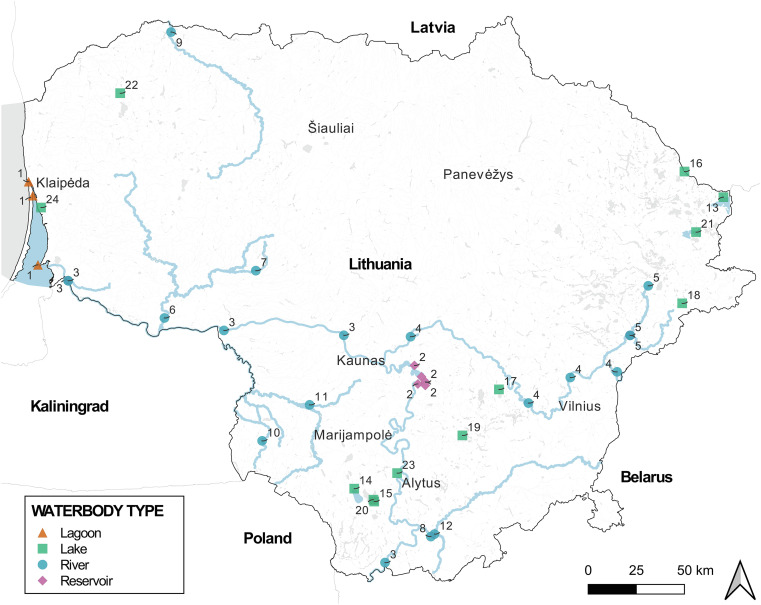
Map showing fish sampling sites in Lithuania (coordinates of localities are in Table S1). Lagoon: 1 – Curonian Lagoon; Reservoir: 2 – Kaunas reservoir; Rivers: 3 – Nemunas, 4 – Neris, 5 – Žeimena, 6 – Jūra, 7 – Upė, 8 – Merkys, 9 – Venta, 10 – Širvinta, 11 – Pilvė, 12 – Grūda; Lakes: 13 – Drūkšiai, 14 – Dusia, 15 – Maušelis, 16 – Gabris, 17 – Bedugnis, 18 – Šėlinis, 19 – Privalskis, 20 – lake in Statiškės forest, 21 – Dysnai, 22 – swamp near Dvarviečiai, 23 – pond in Alytus, 24 – Gravel pit near Klaipėda.

**Figure 2:**
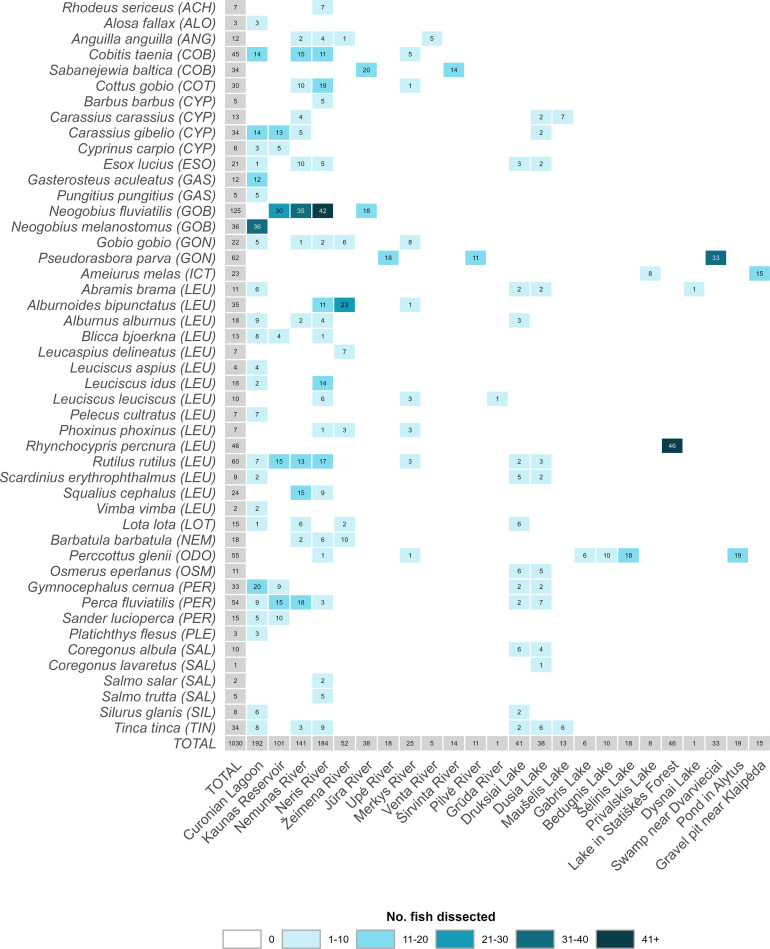
Summary of fish species sampled (in this study, Kudlai *et al.* [43], and Rakauskas *et al.* [70]) and their total number collected in each examined waterbody. Abbreviations: ACH, Acheilognathidae; ALO, Alosidae; ANG, Anguillidae; COB, Cobitidae; COT, Cottidae; CYP, Cyprinidae; ESO, Esocidae; GAS, Gasterosteidae; GOB, Gobiidae; GON, Gobionidae; ICT, Ictaluridae; LEU, Leuciscidae; LOT, Lotidae; NEM, Nemacheilidae; ODO, Odontobutidae; OSM, Osmeridae; PER, Percidae; PLE, Pleuronectidae; SAL, Salmonidae; SIL, Siluridae; TIN, Tincidae.

In total, 1030 fish from 47 species were sampled from 40 localities at 24 waterbodies (10 rivers, 11 lakes, including a water reservoir, 1 pond, 1 swamp, and 1 lagoon) from May to October in 2022–2024 (Figs. 1, 2; Table S1). To ensure compatibility of the newly collected data with historical data from Lithuania, 80% of the same fish species for which helminthological data was previously available were sampled during this study.

Depending on habitat conditions at the sampling localities, fish were collected using several methods, including battery-powered electric fishing gear (Samus Special Electronics, Samus-725 mp), baited crayfish traps (50 cm width and 80 cm length), or beach seine nets (7 m length, 4 mm mesh size). Fish from deep lentic environments were collected with multimesh benthic gillnets, each of which was 40 m in length and 3 m in height. Mesh size varied every 5 m and was 14, 18, 22, 25, 30, 40, 50, and 60 mm.

Sampling was carried out under permits obtained from the Environment Protection Agency, Lithuania (license Nos.: 018 and 019 valid for 2022; 016 and 017 valid for 2023; and 013, 025, and 032 valid for 2024). The collected fish were transported live to a laboratory aquarium and examined within several days after being caught. Fish were identified using the identification key by Kottelat and Freyhof [39]. Fish names and taxonomy followed FishBase [21].

### Dissection of fish hosts and handling of metacercariae

Prior to dissection, fish were individually labelled, measured to the nearest 1 mm, and weighed to the nearest 0.1 g. Thereafter, the external body surface, fins, gills, muscles, and all internal fish organs were separately examined for the presence of helminths under a dissecting microscope. After detecting and collecting helminth specimens, all fish organs were individually pressed between Petri dishes and screened for additional specimens. Detected metacercariae were collected, cleaned from host tissue with fine needles, rinsed in saline solution, and counted. Encysted metacercariae were cold-killed in 80% ethanol, whereas excysted metacercariae were heat-killed in saline solution and thereafter preserved in 80% ethanol.

With some exceptions, metacercarial samples were initially morphologically identified to the family or genus level. However, given the low accuracy of morphological identification of metacercarial stages, specimens representing different morphotypes collected in different hosts from a variety of localities were selected for molecular identification. The morphology of these metacercariae was studied live as wet-mounted specimens with the aid of a compound microscope. A series of photomicrographs was taken for each isolate (photogenophores) using a digital camera AxioCam ERc5s on Zeiss Primo Star light microscope. Photographed specimens were preserved in 96% molecular-grade ethanol. The cysts of encysted metacercariae were broken prior to fixation and their tissue was fixed. The material used in this study is stored in the P.B. Šivickis Laboratory of Parasitology of the State Scientific Research Institute, Nature Research Centre, Lithuania.

### Molecular identification of metacercariae

Specimens of metacercariae preserved for molecular identification were used for DNA extraction by applying a KAPA Express Extract Kit (KAPA Biosystems, Cape Town, South Africa). Partial mitochondrial cytochrome *c* oxidase subunit 1 (*cox*1) sequences were generated for the members of the Bucephalidae, Cyathocotylidae, Diplostomidae, Heterophyidae, Opisthorchiidae, and Strigeidae (483–835 nucleotides), using two sets of primers. Primers Dice1F and Dice14R [94] or primers Dig_cox1Fa and Dig_cox1R [96] were used for amplification following PCR conditions provided in Van Steenkiste *et al.* [94] and Wee *et al.* [96], respectively. The mitochondrial nicotinamide adenine dinucleotide dehydrogenase subunit 1 (*nad*1) sequences (486–501 nt) were generated for members of the Echinochasmidae and Echinostomatidae using NDJ11 and NDJ2A primers [38, 57], following the PCR conditions provided in Laidemitt *et al.* [48]. For most species, the 28S rDNA sequences (1 221–1 313 nt) were generated using the primers ZX-1 [77] and 1500R [91], following the PCR conditions provided in Scholz *et al.* [77]. The sequence of ITS2 (677 nt) was generated using the primers 3S [56] and ITS2.2 [10], following the PCR conditions provided in Cutmore *et al.* [13]. Amplified DNA was verified on a 1% agarose gel and purified with an ExoSAP-IT PCR Cleanup enzymatic kit from Thermo Fisher Scientific, Inc. (Waltham, MA, USA). Sequencing reactions of both strands were carried out using the same PCR primers mentioned above. The newly obtained sequences were revised and assembled using Geneious Prime software ver. 2025.3 (Biomatters, Auckland, New Zealand). Selected novel sequences were deposited in GenBank under accession numbers PX503210, PX549898–PX549899, PX552621–552644, PX552745–PX552809, PX568227–PX568252, PX596715, PX600336–PX600372, PX614069 (Table S2).

To confirm our preliminary identification for sequenced specimens and to select relevant sequences for phylogenetic analyses, a BLAST search for available sequences in GenBank was performed. Sequences obtained in the present study were aligned with selected representative sequences from GenBank using MUSCLE implemented in Geneious Prime. Due to the large amount of newly generated sequences, only those representing a species from its unique host(s) were included in the alignments (Table S2). The sequences of metacercariae recently reported from invasive fish species in Lithuania by Kudlai *et al.* [43] and Rakauskas *et al.* [70] were included in the alignment, and total species counts (*i.e.* number of specimens of each identified species) were provided for a full overview on the current diversity of trematode metacercariae in freshwater fish from Lithuania. Sequences obtained for metacercarial isolates from freshwater fish in Lithuania (present study, Kudlai *et al.* [43], Rakauskas *et al.* [70]) are presented together with their fish hosts on the phylogenetic trees.

A total of six alignments were built and used for phylogenetic analyses, which was performed by applying Bayesian inference (BI) and maximum likelihood (ML) methods. The best-fitting models of nucleotide substitution for the datasets, selected using jModelTest 2.0 [14] based on the Akaike Information Criterion, were GTR + I + G (Alignments of the Cyathocotylidae, Diplostomidae, Echinochasmidae + Echinostomatidae, and Heterophyidae + Opisthorchiidae) and GTR + G (Alignments of the Bucephalidae, and Strigeidae). The ML analysis was conducted using PhyML ver. 3.0 [28] run on the ATGC bioinformatic platform (http://www.atgc-montpellier.fr/phyml/). The BI analysis was conducted using MrBayes software ver. 3.2.3 [75]. Tracer v. 1.6 [72] was used to ensure that effective sample size values for all parameters were above 200 and to determine the burn-in. Finally, output trees were summarised after discarding 25% of generations as burn-in and visualised using FigTree ver. 1.4 software [71]. Sequence divergence used for species delineation was estimated in MEGA ver. 11 [90].

Following the nomenclature of previously published studies that provided numbers for unidentified species, we gave subsequent numbers for the species found in our study.

After molecular identification of selected metacercarial morphotypes, we extrapolated identification to corresponding samples, but often to genus or family level, in order to calculate parasitological indices [prevalence (P%), and intensity of infection (I) presented as min–max (mean) in the text and Table 1] [7]. Due to the enormous number of metacercariae collected, sequencing each specimen to confirm its species identity molecularly was impossible. Therefore, the infection rates in Table 1, which summarises data on metacercariae, their microhabitat within the host, and fish hosts reported in the present study, are provided for either trematode family or genus for all fish hosts. In the few cases where species identification could undoubtedly be confirmed, the infection rates were provided for species of trematode metacercariae. The categories of host specificity proposed by Euzet and Combes [17], *i.e.* oioxenous (infecting a single host species), stenoxenous (infecting closely-related hosts – more than one species of a single family), and euryxenous hosts (infecting unrelated hosts – more than from one family), were used in the present study.

**Table 1:** Summary data of metacercariae, their microhabitat within host, fish hosts, and infection rates reported in the present study.

TF	Trematode species	Microhabitat	Fish hosts	Infection rates
Buc	*Bucephalus polymorphus*	on eye (sclera/cornea), fins, gills, skin, oesophagus, on liver, on intestinal wall, on urinary bladder, muscles	**Acheilognathidae**: *Rhodeus sericeus*^m^; **Cottidae**: *Cottus gobio*; **Gobiidae**: Neogobius fluviatilis*; **Gobionidae**: *Gobio gobio*; **Leuciscidae**: *Abramis brama*, *Alburnoides bipunctatus*, *Alburnus alburnus*, *Blicca bjoerkna*, *Leuciscus idus*, *Leuciscus leuciscus*^m^, *Phoxinus phoxinus*^m^, *Rutilus rutilus*^m^, *Squalius cephalus*, *Vimba vimba*; **Percidae**: *Gymnocephalus cernua*, *Sander lucioperca*^m^	*P* = 19%; *I* = 1–2000 (74)
	*Rhipidocotyle campanula*	in eye, gills, oesophagus, on intestinal wall, on internal organs, muscles	**Gobionidae**: Pseudorasbora parva*; **Leuciscidae**: *Alburnoides bipunctatus*, *Blicca bjoerkna*, *Rutilus rutilus*	
	*Rhipidocotyle fennica*	on eye (sclera/cornea), fins, skin, on liver, on kidney, on intestinal wall, muscles	**Cyprinidae**: *Barbus barbus*; **Gobionidae**: Pseudorasbora parva*; **Leuciscidae**: *Alburnus alburnus*, *Rutilus rutilus*, *Scardinius erythrophthalmus*, *Squalius cephalus*; **Percidae**: *Perca fluviatilis*; **Tincidae**: *Tinca tinca*	
Cya	*Holostephanus dubinini*	fins	**Leuciscidae**: *Leuciscus idus*	*P* = 24%; *I* = 1–1500 (73)
	Cyathocotylidae gen. sp. 1	on eye (sclera/cornea), gills, oesophagus, on heart, on liver, muscles	**Cobitidae**: *Cobitis taenia*, *Sabanejewia baltica*; **Gasterosteidae**: *Pungitius pungitius*; **Gobiidae**: Neogobius fluviatilis*, Neogobius melanostomus*	
	Cyathocotylidae gen. sp. 2	gills, muscles	**Cobitidae**: *Cobitis taenia, Sabanejewia baltica*; **Gobiidae**: Neogobius fluviatilis*	
	Cyathocotylidae gen. sp. 3	gills	**Gobiidae**: Neogobius fluviatilis*	
	Cyathocotylidae gen. sp. 4	fins, skin, muscles	**Leuciscidae**: *Alburnus alburnus*, *Scardinius erythrophthalmus*	
	Cyathocotylidae gen. sp. 5	on eye (sclera/cornea), in eye, vitreous humour, fins, skin, cranial cavity, brain, on internal organs, muscles	**Acheilognathidae**: *Rhodeus sericeus*; **Cyprinidae**: *Barbus barbus*; **Esocidae**: *Esox lucius*; **Gobiidae** Neogobius fluviatilis*; **Gobionidae**: *Gobio gobio*; **Leuciscidae**: *Abramis brama*, *Alburnus alburnus*, *Blicca bjoerkna*, *Leuciscus idus*, *Rutilus rutilus*, *Scardinius erythrophthalmus*, *Squalius cephalus*, *Vimba vimba*; **Nemacheilidae**: *Barbatula barbatula*; **Percidae**: *Sander lucioperca*; **Tincidae**: *Tinca tinca*	
	Cyathocotylidae gen. sp. 6	muscles	**Acheilognathidae**: *Rhodeus sericeus*	
	Cyathocotylidae gen. sp. 7	in eye, vitreous humour, fins, skin, gills, on intestinal wall, on liver, muscles	**Cyprinidae**: *Carassius carassius*, *Carassius gibelio*; **Esocidae**: *Esox lucius*; **Leuciscidae**: *Blicca bjoerkna*, *Leuciscus aspius*, *Pelecus cultratus*, *Scardinius erythrophthalmus*; **Percidae**: *Perca fluviatilis*^m^, *Sander lucioperca*; **Tincidae**: *Tinca tinca*	
Dip	*Diplostomum baeri*	lens	**Leuciscidae**: *Alburnus alburnus*, *Rutilus rutilus*	*P* = 46%; *I* = 1–168 (19)
	*Diplostomum mergi* Lineage 2 of Georgieva *et al.* [23]	lens	**Leuciscidae**: *Abramis brama*, *Alburnoides bipunctatus*, *Alburnus alburnus, Blicca bjoerkna*, *Leuciscus leuciscus*, *Rutilus rutilus*, *Vimba vimba*	
	*Diplostomum parviventosum*	lens	**Leuciscidae**: *Alburnoides bipunctatus*; **Gobionidae**: *Gobio gobio*	
	*Diplostomum pseudospathaceum*	lens	**Cottidae**: *Cottus gobio*; **Cyprinidae**: *Carassius gibelio*; **Esocidae**: *Esox lucius;* **Gasterosteidae**: *Gasterosteus aculeatus*; **Gobiidae**: Neogobius fluviatilis*, Neogobius melanostomus*; **Gobionidae**: *Gobio gobio*; **Ictaluridae**: Ameiurus melas*; **Leuciscidae**: *Abramis brama*, *Alburnoides bipunctatus*, *Alburnus alburnus*, *Blicca bjoerkna*, *Leucaspius delineatus*, *Leuciscus aspius*,	
			*Leuciscus idus*, *Leuciscus leuciscus*, *Scardinius erythrophthalmus*, *Squalius cephalus*; **Lotidae**: *Lota lota*; **Nemacheilidae**: *Barbatula barbatula*; **Percidae**: *Gymnocephalus cernua*, *Sander lucioperca*; **Salmonidae**: *Coregonus albula*, *Salmo salar*; **Siluridae**: *Silurus glanis*; **Tincidae**: *Tinca tinca*	
	*Diplostomum spathaceum*	lens	**Alosidae**: *Alosa fallax*; **Anguillidae**: *Anguilla anguilla*; **Cyprinidae**: *Barbus barbus, Carassius carassius*, *Carassius gibelio, Cyprinus carpio;* **Gasterosteidae**: *Gasterosteus aculeatus*; **Gobiidae**: Neogobius fluviatilis*, Neogobius melanostomus*; **Gobionidae**: *Gobio gobio*, Pseudorasbora parva*; **Ictaluridae**: Ameiurus melas*; **Leuciscidae**: *Abramis brama*, *Blicca bjoerkna*, *Leuciscus aspius*, *Leuciscus idus*, *Pelecus cultratus*, *Rutilus rutilus*, *Scardinius erythrophthalmus*; **Lotidae**: *Lota lota*; **Osmeridae**: *Osmerus eperlanus*; **Percidae**: *Gymnocephalus cernua*, *Perca fluviatilis*, *Sander lucioperca*; **Pleuronectidae**: *Platichthys flesus*; **Siluridae**: *Silurus glanis*	
	*Diplostomum* sp. A of Kudlai *et al.* [38]	lens	**Cyprinidae**: *Carassius gibelio*; **Tincidae**: *Tinca tinca*	
	*Diplostomum* sp. C of Kudlai *et al.* [38]	lens	**Leuciscidae**: *Scardinius erythrophthalmus*	
	*Diplostomum* sp. Lineage 3 of Blasco-Costa *et al.* [5]	vitreous humour, lens	**Cottidae**: *Cottus gobio*; **Esocidae**: *Esox lucius* **Leuciscidae**: *Alburnus alburnus*, *Leuciscus idus*, *Leuciscus leuciscus*, *Rutilus rutilus*, *Scardinius erythrophthalmus*, *Squalius cephalus*; **Salmonidae**: *Coregonus albula*	
	*Diplostomum* sp. Lineage 4 of Blasco-Costa *et al.* [5]	retina, lens, vitreous humour	**Lotidae**: *Lota lota*; **Percidae**: *Gymnocephalus cernua*, *Perca fluviatilis*	
	*Diplostomum* sp. Lineage 5 of Blasco-Costa *et al.* [5]	retina	**Osmeridae**: *Osmerus eperlanus*; **Salmonidae**: *Coregonus albula*	
	*Diplostomum* sp. Lineage 6 of Blasco-Costa *et al.* [5]	lens, retina	**Gasterosteidae**: *Pungitius pungitius*	
	*Hysteromorpha triloba*	muscles, gills	**Leuciscidae**: *Abramis brama*, *Alburnus alburnus*, *Blicca bjoerkna*^m^*, Leuciscus aspius*^m^*, Leuciscus idus*^m^, *Rutilus rutilus*, *Vimba vimba*^m^; **Tincidae**: *Tinca tinca*	*P* = 3.4%; *I* = 1–164 (18)
	*Posthodiplostomum brevicaudatum*	in eye, vitreous humour	**Esocidae**: *Esox lucius*; **Leuciscidae**: *Rutilus rutilus*, *Scardinius erythrophthalmus*; **Percidae**: *Perca fluviatilis*	*P* = 1.2%; *I* = 1–43 (7)
	*Posthodiplostomum cuticola*	skin, fins, gills, on intestinal wall, muscles	**Cobitidae**: *Cobitis taenia*; **Cyprinidae**: *Carassius gibelio*^m^; **Gobiidae**: Neogobius fluviatilis*; **Leuciscidae**: *Abramis brama*, *Alburnoides bipunctatus*, *Alburnus alburnus*, *Blicca bjoerkna*, *Leuciscus aspius*, *Leuciscus idus*^m^, *Leuciscus leuciscus*, *Pelecus cultratus*^m^, *Rutilus rutilus*^m^, *Scardinius erythrophthalmus*^m^	*P* = 4.5%; *I* = 1–33 (5)
	*Posthodiplostomum scardinii*	brain	**Leuciscidae**: *Abramis brama*, *Leuciscus idus*, *Scardinius erythrophthalmus*, *Squalius cephalus*	*P* = 1.4%; *I* = 1–245 (34)
	*Tylodelphys clavata*	vitreous humour, cranial cavity	**Cyprinidae**: *Carassius gibelio*^m^, *Cyprinus carpio*; **Esocidae**: *Esox lucius*^m^; **Gobiidae**: Neogobius fluviatilis*, Neogobius melanostomus*; **Leuciscidae**: *Abramis brama*^m^, *Alburnus alburnus*, *Blicca bjoerkna*, *Leuciscus aspius*^m^, *Leuciscus idus*^m^, *Pelecus cultratus*^m^, *Rutilus rutilus, Squalius cephalus*, *Scardinius erythrophthalmus*^m^, *Vimba vimba*^m^; **Lotidae**: *Lota lota*; **Percidae**: *Gymnocephalus cernua, Perca fluviatilis*, *Sander lucioperca*; **Pleuronectidae**: *Platichthys flesus*^m^; **Salmonidae**: *Coregonus albula*^m^; **Tincidae**: *Tinca tinca*	
	*Tylodelphys podicipina*	vitreous humour, cranial cavity	**Leuciscidae**: *Rutilus rutilus*^m^; **Lotidae**: *Lota lota*; **Percidae**: *Gymnocephalus cernua*, *Perca fluviatilis*^m^	*P* = 17% ; *I* = 1–155 (22)
	*Tylodelphys* sp.	vitreous humour	**Gobionidae**: Pseudorasbora parva*; **Salmonidae**: *Salmo salar*	
Ech	*Echinochasmus* sp. 1	gills	**Percidae**: *Perca fluviatilis*	*P* = 0.2%; *I* = 1–3 (2)
Echi	*Echinoparyphium* sp. 2	kidney	**Cobitidae**: *Cobitis taenia*, *Sabanejewia baltica*	*P* = 1.8%; *I* = 1–109 (24)
	*Petasiger phalacrocoracis*	gills	**Cobitidae**: *Cobitis taenia*; **Tincidae**: *Tinca tinca*	*P* = 0.4%; *I* = 1
	*Petasiger radiatus*	on eye (sclera/cornea)	**Cyprinidae**: *Carassius gibelio*	*P* = 0.1%; *I* = 1
Het	*Apophallus donicus*	skin, fins, gills, in eye, brain, in heart	**Cottidae**: *Cottus gobio*; **Esocidae**: *Esox lucius*; **Percidae**: *Gymnocephalus cernua*, *Perca fluviatilis*, *Sander lucioperca*	*P* = 18%; *I* = 1–3174 (178)
	*Apophallus muehlingi*	skin, fins, in eye, lens, muscles	**Gobionidae**: *Gobio gobio*; **Leuciscidae**: *Alburnoides bipunctatus*, *Alburnus alburnus*, *Blicca bjoerkna*, *Scardinius erythrophthalmus*, *Vimba vimba*	
	*Apophallus* sp.	skin, fins, gills, on eye (sclera/cornea), in eye, vitreous humour, muscles	**Cyprinidae**: *Barbus barbus*, *Carassius gibelio*; **Esocidae**: *Esox lucius*; **Cobitidae**: *Sabanejewia baltica*^m^; **Gobiidae**: Neogobius fluviatilis*; **Gobionidae**: *Gobio gobio*; **Leuciscidae**: *Abramis brama*, *Leuciscus leuciscus*, *Pelecus cultratus*, *Phoxinus phoxinus*, *Rutilus rutilus*, *Scardinius erythrophthalmus*, *Squalius cephalus*; **Lotidae**: *Lota lota*; **Nemacheilidae**: *Barbatula barbatula*; **Salmonidae**: *Salmo trutta*; **Siluridae**: *Silurus glanis*; **Tincidae**: *Tinca tinca*	
Opi	*Metorchis bilis*	fins	**Leuciscidae**: *Leuciscus idus*	*P* = 0.9%; *I* = 1–50 (12)
	*Metorchis* sp. 1	on internal organs, muscles	**Cobitidae**: *Cobitis taenia*, *Sabanejewia baltica*	
	*Metorchis* sp. 2	on eye (sclera/cornea), muscles	**Leuciscidae**: *Rutilus rutilus*; **Tincidae**: *Tinca tinca*	
Str	*Apatemon gracilis*	in eye, on intestinal wall	**Nemacheilidae**: *Barbatula barbatula*; **Percidae**: *Perca fluviatilis*	*P* = 16%; *I* = 1–600 (24)
	*Apatemon* sp. 7	gills, brain, on heart and intestinal wall	**Gobiidae**: Neogobius fluviatilis*	
	*Apatemon* sp. 8	in eye	**Gobiidae**: Neogobius fluviatilis*, Neogobius melanostomus*	
	*Ichthyocotylurus* sp. 2	muscles, brain, on eye, on gills and internal organs (kidney, gonads, swim bladder)	**Percidae**: *Gymnocephalus cernua*, *Perca fluviatilis*, *Sander lucioperca*	
				
	*Ichthyocotylurus* sp. 4	on internal organs (heart, intestinal wall)	**Leuciscidae**: *Abramis brama*, *Alburnus alburnus*^m^, *Blicca bjoerkna*, *Leuciscus aspius*, *Pelecus cultratus*, *Scardinius erythrophthalmus*, *Vimba vimba*; **Percidae**: *Sander lucioperca*	
	*Ichthyocotylurus* sp. 5	on internal organs (heart, liver, intestinal wall)	**Gobionidae**: *Gobio gobio*; **Esocidae**: *Esox lucius*; **Leuciscidae**: *Leuciscus idus*; **Percidae**: *Gymnocephalus cernua*, *Sander lucioperca*; **Pleuronectidae**: *Platichthys flesus*	
	*Ichthyocotylurus* sp. 6	on internal organs	**Osmeridae**: *Osmerus eperlanus*	
	*Ichthyocotylurus* sp. 7	on internal organs (heart, urinary bladder, intestinal wall)	**Percidae**: *Perca fluviatilis*	
	*Ichthyocotylurus* sp. 8	on internal organs (heart)	**Salmonidae**: *Coregonus albula*	
	*Ichthyocotylurus* sp. 9	on internal organs (intestinal wall)	**Esocidae**: *Esox lucius*	
	Strigeidae gen. sp. 1	on internal organs (intestinal wall)	**Cyprinidae**: *Carassius carassius*, *Carassius gibelio*, *Cyprinus carpio*	
	Strigeidae gen. sp. 2	muscles	**Leuciscidae**: *Leucaspius delineatus*	

Abbreviations: TF, trematode family; Buc, Bucephalidae; Cya, Cyathocotylidae; Dip, Diplostomidae; Ech, Echinochasmidae; Echi, Echinostomatidae; Het, Heterophyidae; Opi, Opisthorchiidae; Str, Strigeidae.

m Species of metacercariae from this fish host was morphologically identified.

Fish species without superscript were molecularly confirmed as host for corresponding species of trematode metacercariae.

* Data (molecularly confirmed) from Kudlai *et al.* [43] and Rakauskas *et al.* [70].

### Dataset and analysis

To compare data obtained in the present study to historical data embedded in the literature, a database of records of trematode species reported as metacercaria from freshwater fish in Lithuania was created. Most records were derived from Rauckis [73], with additional records added from more recent papers of Stankus [85], Bacevičius [3], Rakauskas *et al.* [69], Rakauskas and Blaževičius [67, 68], and Mažeika *et al.* [54]. However, each of the latter papers reported on metacercariae of eight species [*Apophallus muehlingi* (Jägerskiöld, 1899), *Diplostomum spathaceum* (Rudolphi, 1819), *Ichthyocotylurus pileatus* (Rudolphi, 1802), *I. platycephalus* (Creplin, 1825), *I. variegatus* (Creplin, 1825), *Paracoenogonimus ovatus* Katsurada, 1914, *Posthodiplostomum cuticola* (von Nordmann, 1832), and *Tylodelphys clavata* (von Nordmann, 1832)] – all previously reported by Rauckis [73]. The taxonomy of trematode species was verified and, when required, modified following the latest updates. Aside from the phylogenetic trees and metacercariae photomicrographs, all other visualisations were generated within the R statistical environment (R version 4.4.2; R Core Team [66]).

## Results

Out of 1030 examined fish (data from present study, Kudlai *et al.* [43], and Rakauskas *et al.* [70]), belonging to 47 species of 21 families sampled from 24 waterbodies (Fig. 2), 737 individuals of 44 species from 18 water bodies contained metacercariae (*P* = 72%), often at great numbers (Table 1); the number of metacercariae sometimes reached extremely high values (over 3000). Overall, infection with metacercariae was highly variable between different fish species (and individuals) and waterbodies. The highest prevalence of infection was detected in fish from the Ictaluridae (100%), Percidae (98%), Tincidae (88%), Esocidae (86%), and Cobitidae (84%), whereas the lowest prevalence was detected in the Anguillidae (8%), Nemacheilidae (22%), and Cottidae (23%). Considering only waterbodies where more than one fish species was examined, the highest prevalence of infection with metacercariae was reported in fish from Druksiai Lake (95%), Curonian Lagoon (92%), and Kaunas reservoir (90%). Fish from the family Leuciscidae harboured the highest number of trematode species at the metacercarial stage (27) followed by the Percidae (17), Gobiidae (12), Cyprinidae (11), Gobionidae (11), Esocidae (10), Tincidae (10), Cobitidae (7), Salmonidae (7), Lotidae (6), Cottidae (4), Gasterosteidae (4), Nemacheilidae (4), Acheilognathidae (3), Osmeridae (3), Pleuronectidae (3), Siluridae (3), Ictaluridae (2), Alosidae (1), and Anguillidae (1). Within the family Leuciscidae, two species, *Rutilus rutilus* and *Scardinius erythrophthalmus*, harboured the highest diversity of trematode species (15). The fish species not infected with metacercariae were European whitefish *Coregonus lavaretus* (Salmoniformes: Salmonidae), the Chinese sleeper *Perccottus glenii* (Gobiiformes: Odontobutidae) [43], and lake minnow *Rhynchocypris percnura* (Cypriniformes: Leuciscidae).

**Figure 3:**
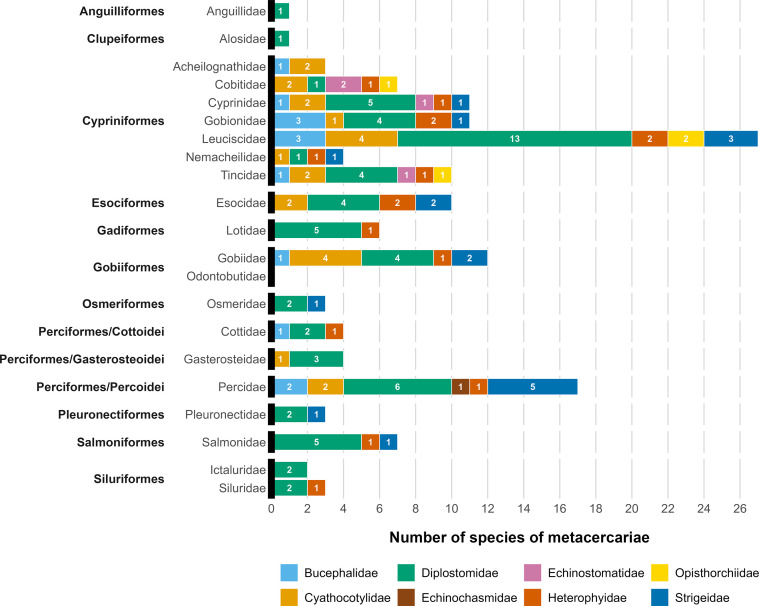
Taxonomic structure of metacercariae in their fish hosts.

A total of 583 sequences (528 *cox*1 and 3 *nad*1 mtDNA, 51 28S rDNA, and 1 ITS2) were generated for 538 metacercarial isolates to verify morphological identification and provide identification to the lowest possible taxonomic level. Both comparative sequence and phylogenetic analyses revealed the presence of metacercariae of 51 species from the families Diplostomidae (18 species), Strigeidae (12), Cyathocotylidae (8), Bucephalidae (3), Echinostomatidae (3), Heterophyidae (3), Opisthorchiidae (3), and Echinochasmidae (1) (Table 1). Twenty-one species were identified to the species level, 21 to the genus level, and nine to the family level. Half of the examined fish were infected with metacercariae from the family Diplostomidae (50%) and almost one quarter were infected with metacercariae from the family Cyathocotylidae (24%). Members of the Bucephalidae were found in 19% of examined fish, followed by the Heterophyidae (18%) and Strigeidae (16%). Metacercariae of the Echinostomatidae (1.8%), Opisthorchiidae (0.9%), and Echinochasmidae (0.2%) were rarely found (Fig. 3).

Fish were often infected with metacercariae from multiple families. Out of 737 infected fish, 332 (45%) specimens were infected with metacercariae from a single family, whereas 405 (55%) specimens were infected with metacercariae from multiple families [two families – 245 (33%), three families – 116 (16%), four families – 39 (5%), and five families – 5 (1%)].

The taxonomic structure of metacercariae varied between different families of fish hosts (Fig. 3). The parasite fauna of the members of the Leuciscidae was not only characterised by the highest species richness, but also the highest taxonomic representation of trematodes, together with the Cyprinidae, Percidae, and Tincidae (6 trematode families per fish family).

Details on each recorded trematode family are listed below in alphabetic order.

### Family Bucephalidae

**Figure 4:**
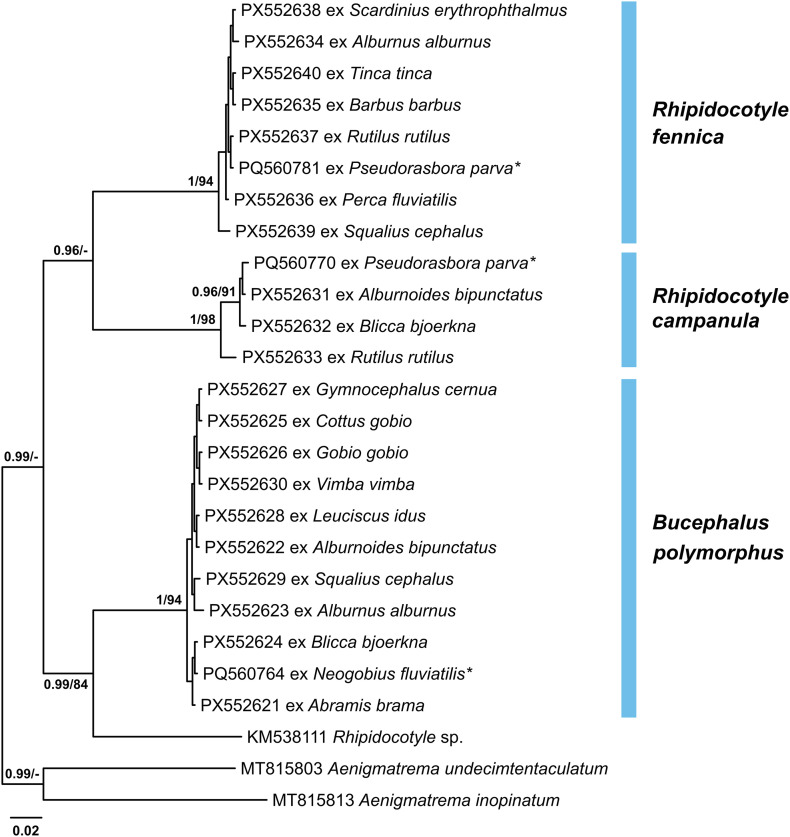
Bayesian inference (BI) and maximum likelihood (ML) phylogram reconstructed using the partial *cox*1 sequences for species of the Bucephalidae. Nodal support from BI and ML analyses indicated as BI/ML; only values > 0.90 (BI) and > 70 (ML) are displayed. The scale-bar indicates expected number of substitutions per site. Novel sequences are shown as the GenBank accession number followed by fish host name. Names of trematode species presented next to coloured vertical bars. Sequences from Kudlai *et al.* [43] are indicated by stars.

**Figure 5:**
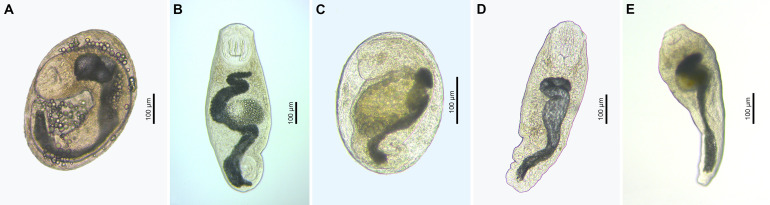
Microphotographs of metacercariae (live) of the family Bucephalidae. (A) *Bucephalus polymorphus*, encysted metacercaria ex *Gobio gobio* (voucher, FP493, see Table S2); (B) *B. polymorphus*, excysted metacercaria ex *Vimba vimba* (voucher, PX552630); (C) *Rhipidocotyle fennica*, encysted metacercaria ex *Squalius cephalus* (voucher, PX552639); (D) *R. fennica*, excysted metacercaria ex *Scardinius erythrophthalmus* (voucher, PX552638); (E) *Rhipidocotyle campanula*, excysted metacercaria ex *Blicca bjoerkna* (voucher, PX552632).

Metacercariae of the family Bucephalidae were represented by three species from two genera, namely *Bucephalus polymorphus* von Baer, 1827, *Rhipidocotyle campanula* (Dujardin, 1845), and *Rhipidocotyle fennica* Gibson, Taskinen & Valtonen, 1992 (Figs. 4, 5). They were recorded in various organs of 197 (*P* = 19%) examined fish belonging to the families Acheilognathidae, Cottidae, Cyprinidae, Gobiidae, Gobionidae, Leuciscidae, Percidae, and Tincidae (Table 1). Bucephalid metacercariae were enclosed in thin-walled oval cysts which could be easily broken to excyst metacercariae. The excretory vesicle of metacercariae is tubular and long, filled with granules, and often appeared black in transmitted light. Bucephalid metacercariae exhibit partial progenesis. One of the prominent differences used to distinguish between *Bucephalus polymorphus* and *Rhipidocotyle* spp. is the presence of a rhynchus with seven retractable tentacles in *Bucephalus polymorphus*. However, in the present study, specimens with the retractable tentacles being everted were rarely observed, even in excysted metacercariae. While metacercariae of *Bucephalus polymorphus* and *Rhipidocotyle campanula* were known to parasitise a variety of freshwater fish in Lithuania [54, 73], *Rhipidocotyle fennica* was previously recorded only as an adult from the northern pike *Esox lucius* and cercariae from the duck mussel *Anodonta anatina* [88]. In the present study, the metacercarial stages of this species were found in cyprinid, gobionid, leuciscid, percid, and tincid fishes (Figs. 5C, 5D, Table 1).

### Family Cyathocotylidae

**Figure 6:**
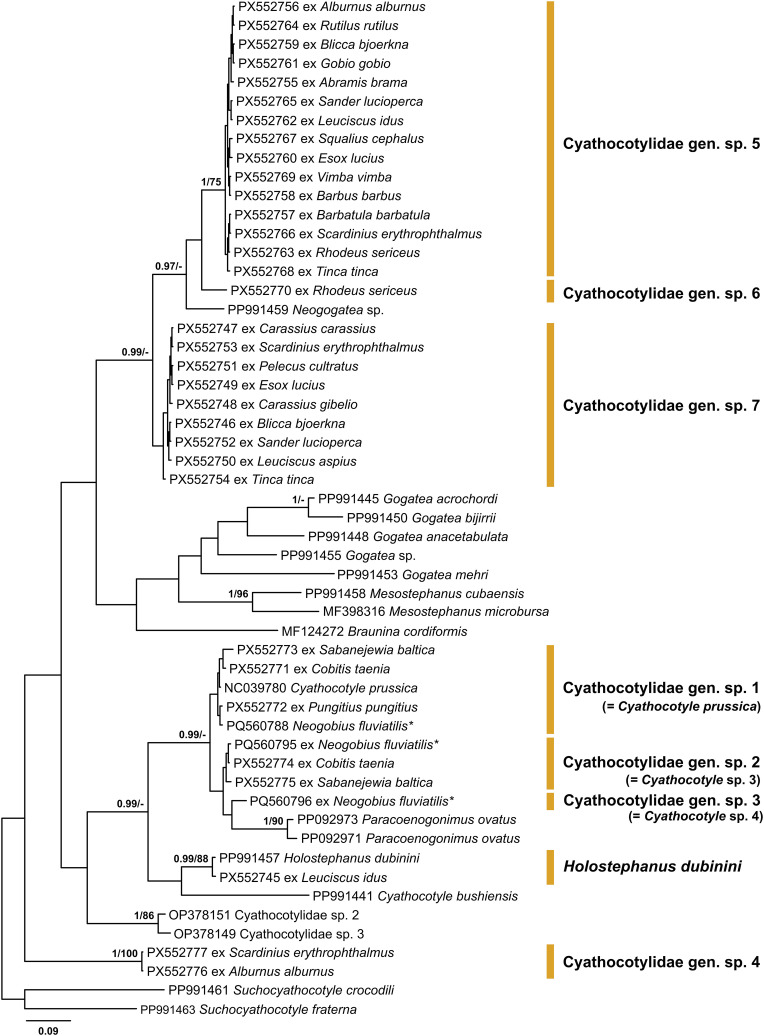
Bayesian inference (BI) and maximum likelihood (ML) phylogram reconstructed using the partial *cox*1 sequences for species of the Cyathocotylidae. Nodal support from BI and ML analyses indicated as BI/ML; only values > 0.90 (BI) and > 70 (ML) are displayed. The scale-bar indicates expected number of substitutions per site. Novel sequences are shown as the GenBank accession number followed by fish host name. Names of trematode species presented next to coloured vertical bars. Sequences from Kudlai *et al.* [43] are indicated by stars.

**Figure 7:**
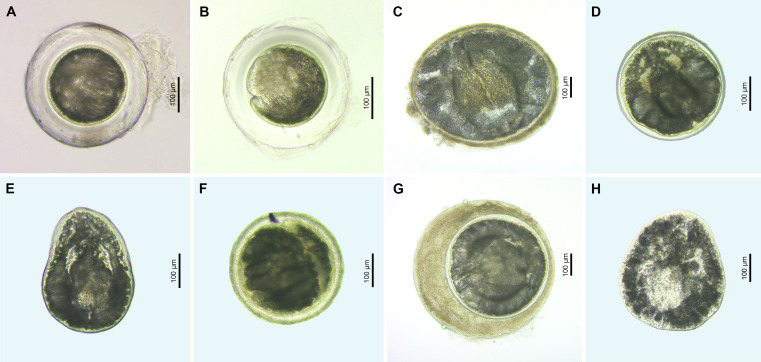
Microphotographs of metacercariae (live) of the family Cyathocotylidae. (A) Cyathocotylidae gen. sp. 1, encysted metacercaria ex *Pungitius pungitius* (voucher, PX552772); (B) Cyathocotylidae gen. sp. 2, encysted metacercaria ex *Cobitis taenia* (voucher, FP535, see Table S2); (C) Cyathocotylidae gen. sp. 4, encysted metacercaria ex *Alburnus alburnus* (voucher, PX552776); (D) Cyathocotylidae gen. sp. 5, encysted metacercaria ex *Abramis brama* (voucher, PX552755); (E) Cyathocotylidae gen. sp. 5, excysted metacercaria ex *Alburnus alburnus* (voucher, PX552756); (F) Cyathocotylidae gen. sp. 6, encysted metacercaria ex *Rhodeus sericeus* (voucher, PX552770); (G) Cyathocotylidae gen. sp. 7, encysted metacercaria ex *Tinca tinca* (voucher, FP1111, see Table S2); (H) Cyathocotylidae gen. sp. 7, excysted metacercaria ex *Sander lucioperca* (voucher, PX552752).

A total of eight species of the Cyathocotylidae were recognised (Figs. 6, 7); however, they were initially identified as belonging to three morphotypes. Most cyathocotylid metacercariae were found in muscle tissue, but they were also present on the body surface, on eyes (sclera/cornea), in eyes, brain, cranial cavity, gills, and on various other internal organs (Table 1). In total, 24% of examined fish belonging to 11 families were infected with metacercariae of the Cyathocotylidae. Cyathocotylid metacercariae were found to completely fill the space inside their small round cyst, often with a thick external hyalinous layer (Figs. 7A, 7B). Although the excretory granules were numerous, the large round or oval holdfast organ and oral sucker were often visible through the transparent cyst. Molecular analyses showed that the eight species reported in this study belong to at least four genera: *Holostephanus*, three unidentified genera, of which one is possibly *Neogogatea* (Fig. 6), and another unidentified genus recognised as *Cyathocotyle* in several previous studies [6, 19, 43, 80]. However, a recent study by Achatz *et al.* [2] suggested that sequences previously assigned to *Cyathocotyle* should rather be considered members of an unidentified genus. Notably, Cyathocotylidae gen. sp. 5–7, potentially members of the genus *Neogogatea*, were detected as metacercariae in freshwater fish for the first time in Lithuania, and this is possibly the first record of this genus for European fauna. Prior to our study, *Neogogatea* spp. were only known to parasitise birds in North America and Asia [1]. The only species of the Cyathocotylidae previously reported in freshwater fish in Lithuania, *Paracoenogonimus ovatus*, with an occurrence of 0.7–100% [73, 85], was not detected in the present study.

### Family Diplostomidae

**Figure 8:**
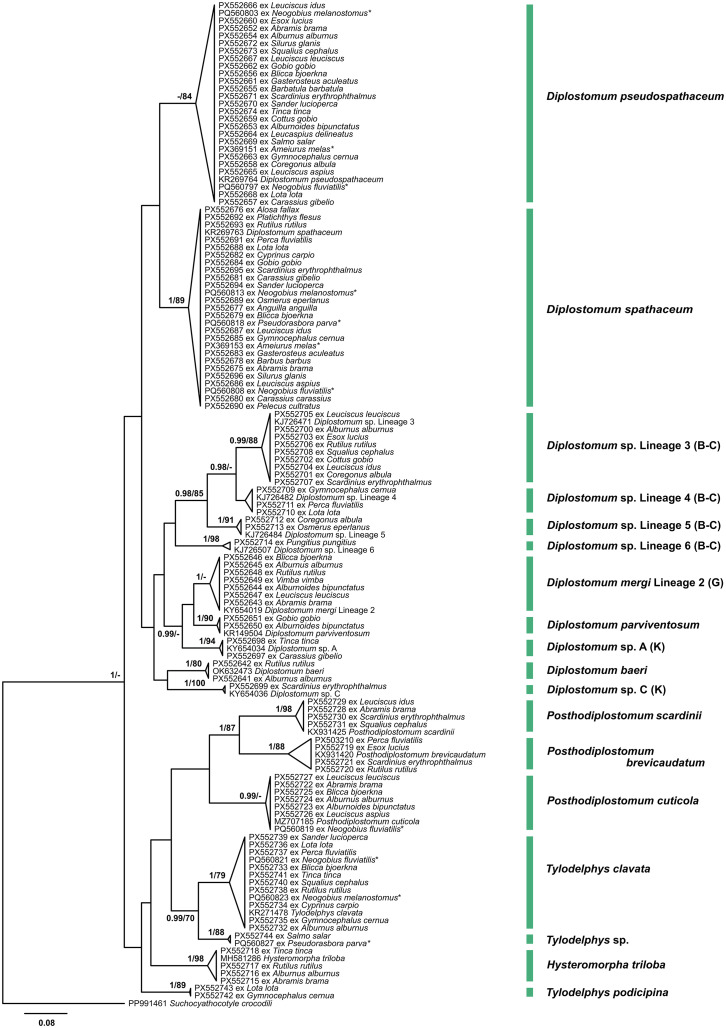
Bayesian inference (BI) and maximum likelihood (ML) phylogram reconstructed using the partial *cox*1 sequences for species of the Diplostomidae. Nodal support from BI and ML analyses indicated as BI/ML; only values > 0.90 (BI) and > 70 (ML) are displayed. The scale-bar indicates expected number of substitutions per site. Novel sequences are shown as the GenBank accession number followed by fish host name. Names of trematode species presented next to coloured vertical bars. Sequences from Kudlai *et al.* [43] and Rakauskas *et al.* [70] are indicated by stars. Abbreviations: B-C, Blasco-Costa *et al.* [5], G, Georgieva *et al.* [23]; K, Kudlai *et al.* [41].

**Figure 9:**
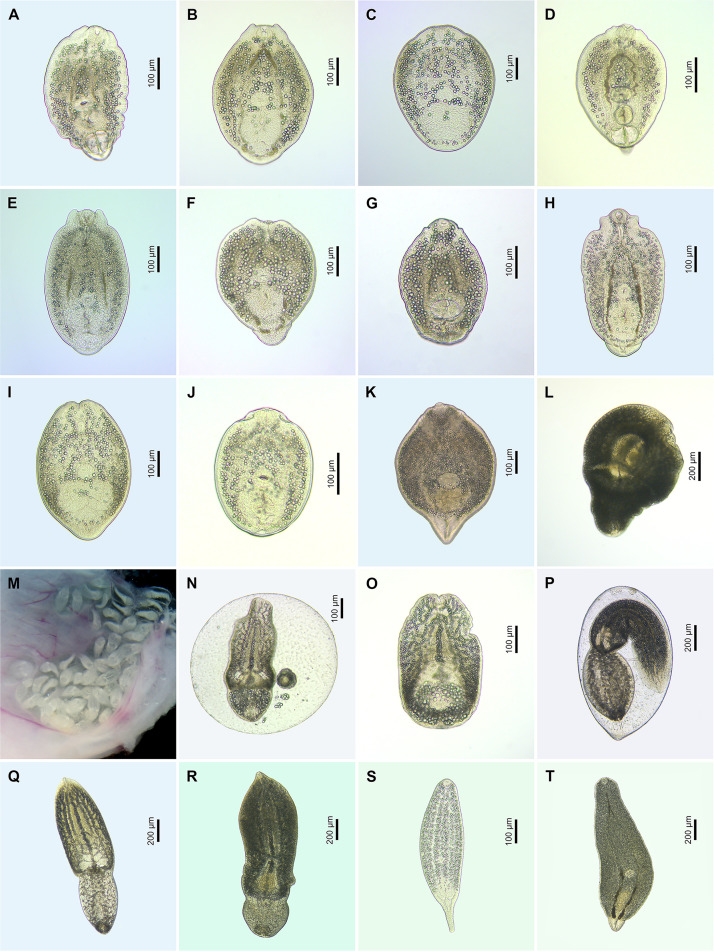
Microphotographs of metacercariae (live) of the family Diplostomidae. (A) *Diplostomum baeri* ex *Rutilus rutilus* (voucher, FP1150, see Table S2); (B) *D. mergi* Lineage 2 of Georgieva *et al.* [23] ex *Abramis brama* (voucher, FP1190, see Table S2); (C) *D. parviventosum* ex *Gobio gobio* (voucher, FP542, see Table S2); (D) *D. pseudospathaceum* ex *Silurus glanis* (voucher, PX552672); (E) *D. spathaceum* ex *Cyprinus carpio* (voucher, PX552682); (F) *Diplostomum* sp. A of Kudlai *et al.* [41] ex *Carassius gibelio* (voucher, PX552697); (G) *Diplostomum* sp. C of Kudlai *et al.* [41] ex *Scardinius erythrophthalmus* (voucher, PX552699); (H) *Diplostomum* sp. Lineage 3 of Blasco-Costa *et al.* [5] ex *S. erythrophthalmus* (voucher, FP702, see Table S2); (I) *Diplostomum* sp. Lineage 4 of Blasco-Costa *et al.* [5] ex *Perca fluviatilis* (voucher, FP673, see Table S2); (J) *Diplostomum* sp. Lineage 5 of Blasco-Costa *et al.* [5] ex *Coregonus albula* (voucher, PX552712); (K) *Diplostomum* sp. Lineage 6 of Blasco-Costa *et al.* [5] ex *Pungitius pungitius* (voucher, PX552714); (L) *Hysteromorpha triloba*, excysted metacercaria ex *Tinca tinca* (voucher, PX552718); (M) *Posthodiplostomum scardinii* inside of the brain tissue ex *S. erythrophthalmus*; (N) *P. scardinii*, encysted metacercaria ex *S. erythrophthalmus* (voucher, PX552730); (O) *P. scardinii*, excysted metacercaria ex *S. erythrophthalmus* (voucher, PX552730); (P) *Posthodiplostomum brevicaudatum*, encysted metacercaria ex *S. erythrophthalmus* (voucher, PX552721); (Q) *P. brevicaudatum*, excysted metacercaria ex *Esox lucius* (voucher, FP1110, see Table S2); (R) *Posthodiplostomum cuticola*, excysted metacercaria ex *Abramis brama* (voucher, PX552722); (S) *Tylodelphys clavata* ex *P*. *fluviatilis*; (T) *Tylodelphys podicipina* ex *Lota lota* (voucher, FP1156, see Table S2).

With 18 species from seven genera, the Diplostomidae was the most diverse family in our material (Figs. 8, 9, Table 1). They were found in 40 out of 47 examined fish species (85%). Among diplostomid metacercariae, the genus *Diplostomum* was represented by 11 species, which were recorded in almost half (46%) of examined fish. Most of the detected *Diplostomum* spp. were lens-infecting (7 species). The second most common diplostomid metacercariae, *Tylodelphys* spp. were mainly found in the vitreous humour and rarely in the cranial cavity. Although the genus *Tylodelphys* was represented by three species, *Tylodelphys clavata* was most commonly found, with its host spectrum being much wider compared to its congeners, *Tylodelphys podicipina* Kozicka & Niewiadomska, 1960 and *Tylodelphys* sp. (Table 1). Metacercariae of *Hysteromorpha triloba* (Rudolphi, 1819) and *Posthodiplostomum* spp. were recorded less often, being found in different fish organs (Table 1). While metacercariae of *Diplostomum* and *Tylodelphys* do not form cysts, metacercariae of *Hysteromorpha triloba* and *Posthodiplostomum* spp. appeared within large thin-walled cysts that could easily be broken to excyst metacercariae. Metacercariae of *Posthodiplostomum cuticola* were additionally enclosed in black capsules. Encysted metacercariae of *Posthodiplostomum scardinii* (Shulman, 1952) were often enclosed in large thin-walled capsules. Based on previous studies conducted in Lithuania, diplostomid metacercariae were the most diverse in freshwater fish, with 15 species reported [3, 54, 65, 67–69, 73, 85].

### Family Echinochasmidae

**Figure 10:**
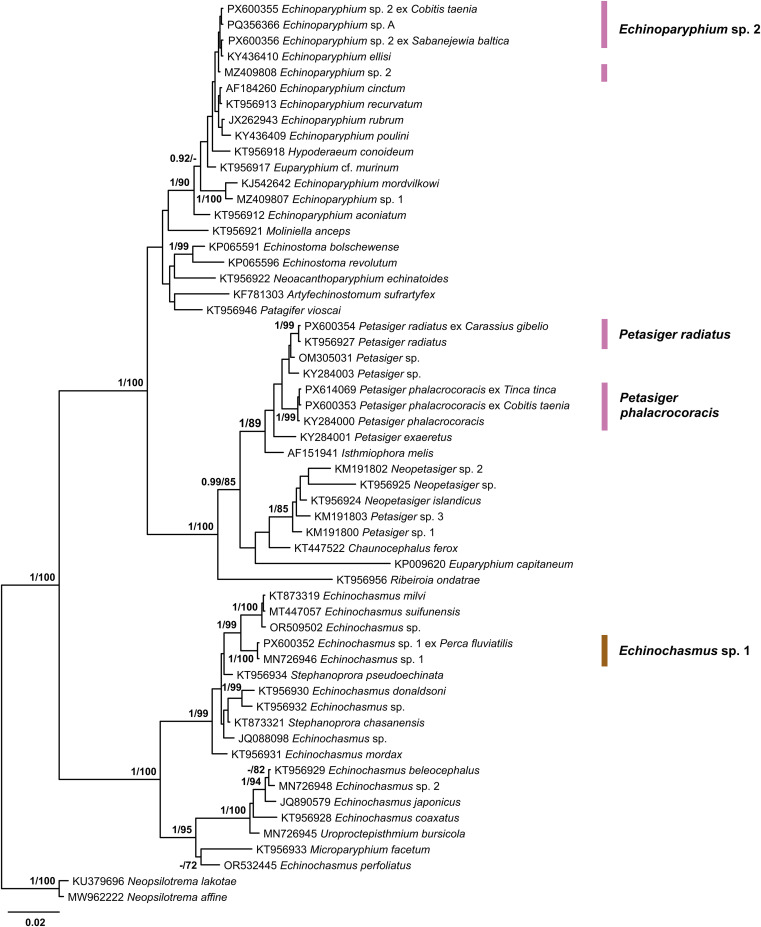
Bayesian inference (BI) and maximum likelihood (ML) phylogram reconstructed using the partial 28S rDNA sequences for species of the Echinochasmidae and Echinostomatidae. Nodal support from BI and ML analyses indicated as BI/ML; only values > 0.90 (BI) and > 70 (ML) are displayed. The scale-bar indicates expected number of substitutions per site. Novel sequences are shown as the GenBank accession number followed by species name and fish host name. Names of trematode species presented next to coloured vertical bars.

**Figure 11:**
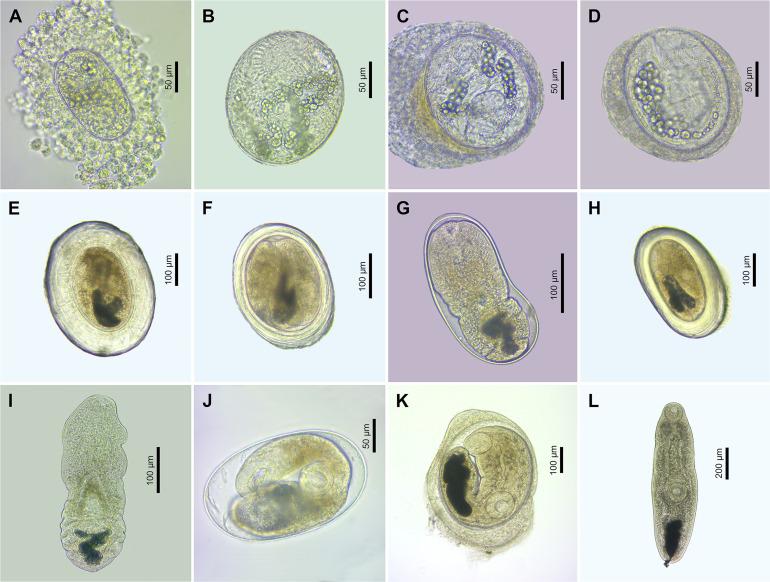
Microphotographs of metacercariae (live) of the families Echinochasmidae (A), Echinostomatidae (B–D), Heterophyidae (E–I) and Opisthorchiidae (J–L). (A) *Echinochasmus* sp. 1, encysted ex *Perca fluviatilis* (voucher, PX600352); (B) *Echinoparyphium* sp. 2, encysted metacercaria ex *Cobitis taenia*; (C) *Petasiger phalacrocoracis*, encysted metacercaria ex *Tinca tinca* (voucher, FP1121, see Table S2); (D) *Petasiger radiatus*, encysted metacercaria ex *Carassius gibelio* (voucher, PX600354); (E) *Apophallus donicus*, encysted metacercaria ex *Esox lucius* (voucher, FP651, see Table S2); (F) *Apophallus muehlingi*, encysted metacercaria ex *Blicca bjoerkna* (voucher, PX552785); (G) *Apophallus muehlingi*, encysted metacercaria ex *Scardinius erythrophthalmus* (voucher, PX552787); (H) *Apophallus* sp., encysted metacercaria ex *S. erythrophthalmus* (voucher, PX552801); (I) *Apophallus* sp., excysted metacercaria ex *Tinca tinca* (voucher, PX552804); (J) *Metorchis bilis*, encysted metacercaria ex *Leuciscus idus* (voucher, PX552805); (K) *Metorchis* sp. 2, encysted metacercaria ex *T. tinca* (voucher, FP1206, see Table S2); (L) *Metorchis* sp. 2, excysted metacercaria ex *Rutilus rutilus* (voucher, PX552808).

A single species of the family Echinochasmidae, *Echinochasmus* sp. 1, was recorded in two specimens of the European perch *Perca fluviatilis*, with low intensity of infection (Figs. 10, 11A, Table 1). Metacercariae were encysted in small thin-walled, transparent, oval cysts. The collar spines and large excretory granules were visible. The affinity of the metacercariae to the genus *Echinochasmus* was confirmed based on the results of our molecular analysis. The newly generated 28S rDNA sequence was identical to sequences of *Echinochasmus* sp. 1 obtained from cercariae recorded in *Bithynia tentaculata* collected in the Curonian Lagoon by Schwelm *et al.* [80]. Echinochasmid metacercariae were not previously reported in freshwater fish from Lithuania, but representatives of this genus were reported from molluscs and birds [27, 37, 80, 83, 84, 89, 95].

### Family Echinostomatidae

Similar to echinochasmid, echinostome metacercariae were rarely found in the fish examined during the present study. Three species from two genera belonging to the family Echinostomatidae were reported, namely *Echinoparyphium* sp. 2, *Petasiger phalacrocoracis* (Yamaguti, 1939), and *P. radiatus* (Dujardin, 1845) (Figs. 10, 11B–11D, Table 1). All three species were reported from fish belonging to different families, with their microhabitat within their hosts differing for each recorded species (Table 1). Echinostome metacercariae were found in small thin-walled, transparent, round cysts often inside a thin-walled transparent capsule. The collar, collar spines, suckers, and large excretory granules were often clearly visible. To date, there were no reports of echinostome metacercariae from freshwater fish in Lithuania. It is worth noting that *Echinoparyphium* sp. 2 is likely a new species to Lithuanian fauna that probably arrived with its invasive intermediate host, *Physella acuta*. It was previously reported from molluscs, namely *Physa gyrina* in North America [26], *P. acuta* in Iceland [61], and recently in *P. acuta* in North America [79]. In the present study, metacercariae of *Echinoparyphium* sp. 2 were found in the kidneys of cobitid fishes, *Cobitis taenia* in the Nemunas River (*P* = 93%, *I* = 1–109) and *Sabanejewia baltica* in the Širvinta River (*P* = 6 out of 6, *I* = 1–28). Interestingly, species of the genus *Echinoparyphium* were reported to use only molluscs as their second intermediate hosts [92]. *Petasiger phalacrocoracis* and *Petasiger radiatus* were previously reported from the great cormorant, *Phalacrocorax carbo*, and molluscs in Lithuania [40, 89].

### Family Heterophyidae

**Figure 12:**
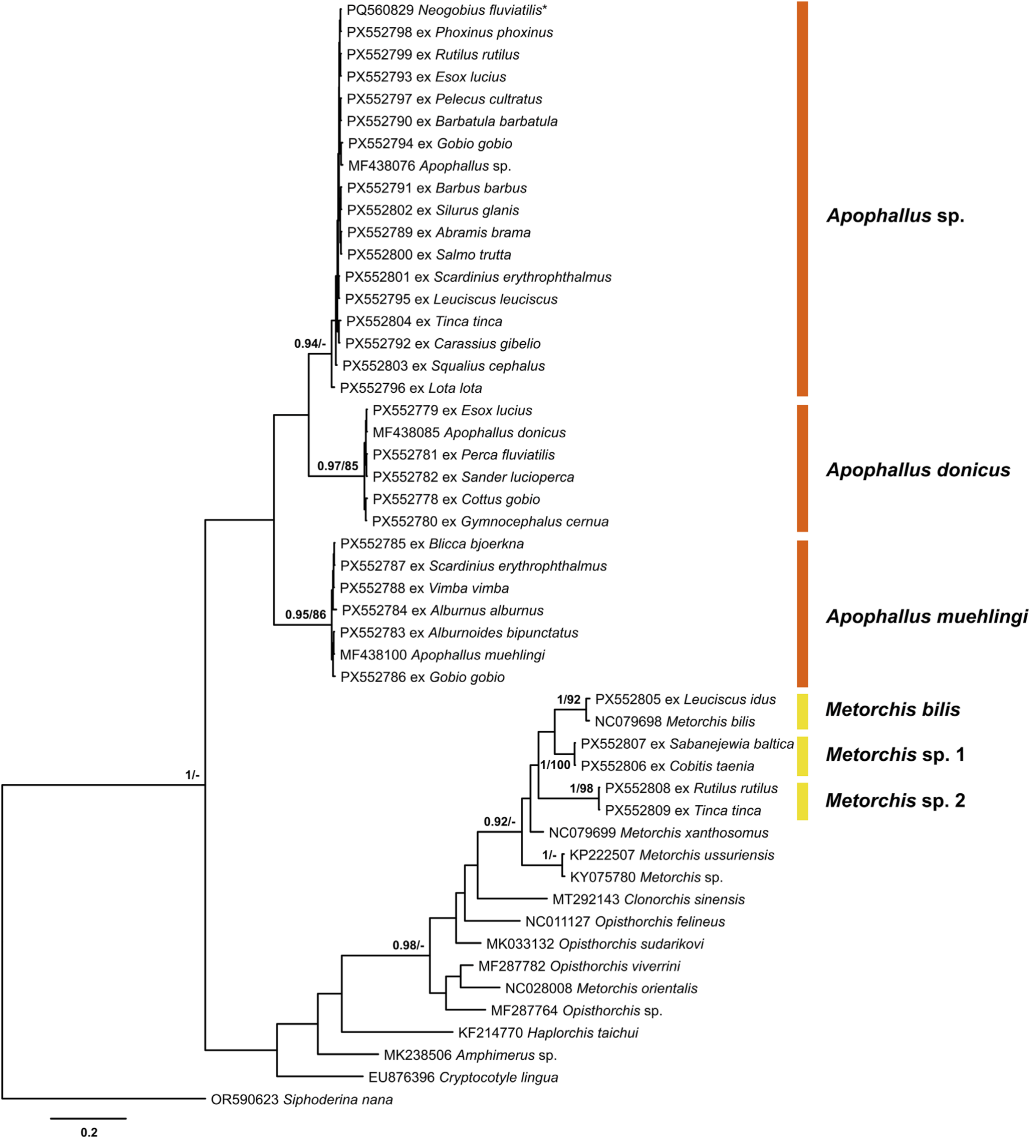
Bayesian inference (BI) and maximum likelihood (ML) phylogram reconstructed using the partial *cox*1 sequences for species of the Heterophyidae and Opisthorchiidae. Nodal support from BI and ML analyses indicated as BI/ML; only values > 0.90 (BI) and > 70 (ML) are displayed. The scale-bar indicates expected number of substitutions per site. Novel sequences are shown as the GenBank accession number followed by fish host name. Names of trematode species presented next to coloured vertical bars. Sequence from Kudlai *et al.* [43] are indicated by star.

Metacercariae of the family Heterophyidae were represented in our material by three species from the genus *Apophallus*, namely *Apophallus donicus* (Skrjabin & Lindtrop, 1919), *Apophallus muehlingi*, and *Apophallus* sp. (Figs. 11E–11I, 12). These metacercariae were mostly found encysted and inside black capsules on fish body surfaces, fins, gills, and rarely in the eyes, brain, heart, and muscles (Table 1). Metacercariae were in most cases enclosed in oval cysts with a very thick hyalinous external layer (Figs. 11E, 11F, 11H). They were actively moving within their cysts. The excretory vesicle is Y-shaped and filled with granules often appearing black in transmitted light. A total of 189 fish individuals (18%) from a variety of families were found to be infected with heterophyid metacercariae (Table 1). Metacercariae of *Apophallus* sp. were the most common among *Apophallus* spp. in our study and were found in a wide range of fish hosts (Table 1). Out of the three heterophyid species found in the present study, *Apophallus muehlingi* was the only species reported in freshwater fish (on fins, gills, and in muscles) in Lithuania prior to our investigation [73, 85].

### Family Opisthorchiidae

Out of three species of the family Opisthorchiidae recorded from five fish species in our study, only one was identified to the species level, *Metorchis bilis* (Braun, 1790) (Fig. 12, Table 1). The two remaining species were only identified to the genus level as *Metorchis* sp. 1 and *Metorchis* sp. 2. Metacercariae of all three species were rarely found in fish during this study (Table 1, Figs. 11J–11L, 12). They were enclosed in thin-walled, round or oval cysts. The suckers, pharynx, caeca, and large dark excretory vesicle were often clearly visible through the transparent cyst wall. To date, metacercariae of a single species of the Opisthorchiidae, *Opisthorchis felineus* (Rivolta, 1884), were reported in freshwater fish from Lithuania, with an occurrence of 6.6–26.6% [73], although this species was surprisingly not recorded in this study. Notably, metacercariae of *Opisthorchis felineus* were recorded from fish belonging to the Cobitidae and Leuciscidae – the same fish families from which *Metorchis* spp. were found in the present study.

### Family Strigeidae

**Figure 13:**
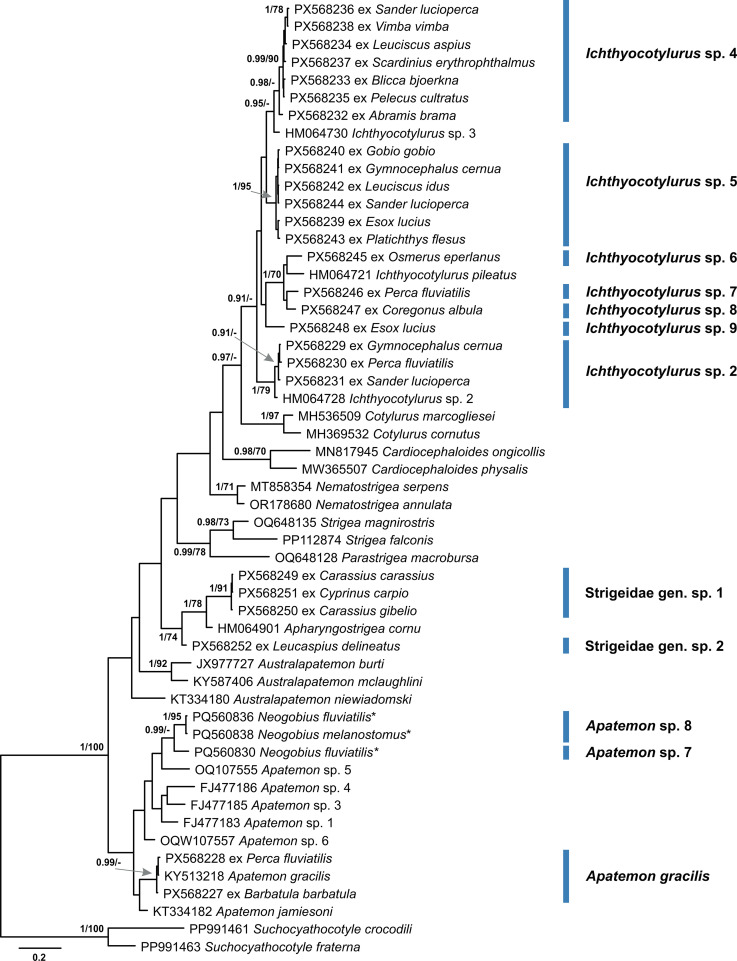
Bayesian inference (BI) and maximum likelihood (ML) phylogram reconstructed using the partial *cox*1 sequences for species of the Strigeidae. Nodal support from BI and ML analyses indicated as BI/ML; only values > 0.90 (BI) and > 70 (ML) are displayed. The scale-bar indicates expected number of substitutions per site. Novel sequences are shown as the GenBank accession number followed by fish host name. Names of trematode species presented next to coloured vertical bars. Sequences from Kudlai *et al.* [43] are indicated by stars.

**Figure 14:**
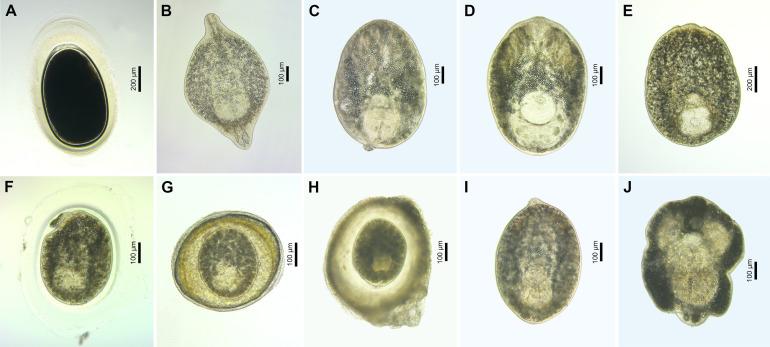
Microphotographs of metacercariae (live) of the families Strigeidae. (A) *Apatemon gracilis*, encysted metacercaria ex *Perca fluviatilis* (voucher, FP440, see Table S2); (B) *A. gracilis*, excysted metacercaria ex *P. fluviatilis* (voucher, PX568228); (C) *Ichthyocotylurus* sp. 2, excysted metacercaria ex *P. fluviatilis* (voucher, FP631, see Table S2); (D) *Ichthyocotylurus* sp. 4, excysted metacercaria ex *Abramis brama* (voucher, PX568232); (E) *Ichthyocotylurus* sp. 5, excysted metacercaria ex *Gymnocephalus cernua* (voucher, PX568241); (F) *Ichthyocotylurus* sp. 6, encysted metacercaria ex *Osmerus eperlanus* (voucher, PX568245); (G) *Ichthyocotylurus* sp. 7, encysted metacercaria ex *P. fluviatilis* (voucher, FP1140, see Table S2); (H) *Ichthyocotylurus* sp. 8, encysted metacercaria ex *Coregonus albula* (voucher, PX568247); (I) *Ichthyocotylurus* sp. 9, excysted metacercaria ex *Esox lucius* (voucher, PX568248); (J) Strigeidae gen. sp. 1, excysted metacercaria ex *Carassius carassius* (voucher, PX568249).

After the Diplostomidae, the Strigeidae was the second most diverse family in the present study. The family was represented by 12 species of which 10 belonged to the genera *Apatemon* and *Ichthyocotylurus*, and two species were identified only to the family level (Figs. 13, 14). The genus *Apatemon* was represented by three species, namely *Apatemon gracilis* (Rudolphi, 1819) found in *Barbatula barbatula* and *Perca fluviatilis* (Table 1), and *Apatemon* sp. 7 and *Apatemon* sp. 8 found in the invasive fish *Neogobius fluviatilis* and *Neogobius melanostomus* [43]. Metacercariae of *Apatemon* were enclosed within egg-shaped cysts with a thick external hyalinous layer (Fig. 14A). The cyst content was always dark and opaque. These metacercariae were rarely found excysted (Figure 9 in Kudlai *et al.* [43]). Metacercariae of *Ichthyocotylurus* were found on a variety of internal organs within their fish hosts (Table 1). They were often enclosed within thin-walled cysts, which could be easily broken to excyst metacercariae and rarely in cysts with a thick hyalinous layer. Sometimes the cysts were additionally enclosed within a thin-walled capsule. Despite numerous granules, the suckers, pseudosuckers, and holdfast organ were often clearly visible. Only one out of seven species of *Ichthyocotylurus* was conspecific with *Ichthyocotylurus* sp. 2 previously reported from *Perca flavescens* in North America [50] (Fig. 13). *Ichthyocotylurus* sp. 4 and *Ichthyocotylurus* sp. 5 were found more often and in a wide range of fish species compared to their congeners *Ichthyocotylurus* spp. 6–9, which were each found from a single host (Table 1). Two unidentified strigeid species, one found on the internal organs of cyprinids, *Carassius carassius*, *Carassius gibelio*, and *Cyprinus carpio*, and another found in the muscle tissue of the leuciscid, *Leucaspius delineatus,* belonged to different genera based on the results of the molecular analysis. Given that metacercariae of only four species of strigeids, all from the genus *Ichthyocotylurus* (*I*. *erraticus* (Rudolphi, 1809), *I*. *pileatus*, *I*. *platycephalus*, and *I*. *variegatus*), have previously been reported from freshwater fishes in Lithuania [54, 67, 68, 73], our study demonstrates that the actual diversity of this family is much higher.

### Changes in diversity and transmission dynamics

**Figure 15:**
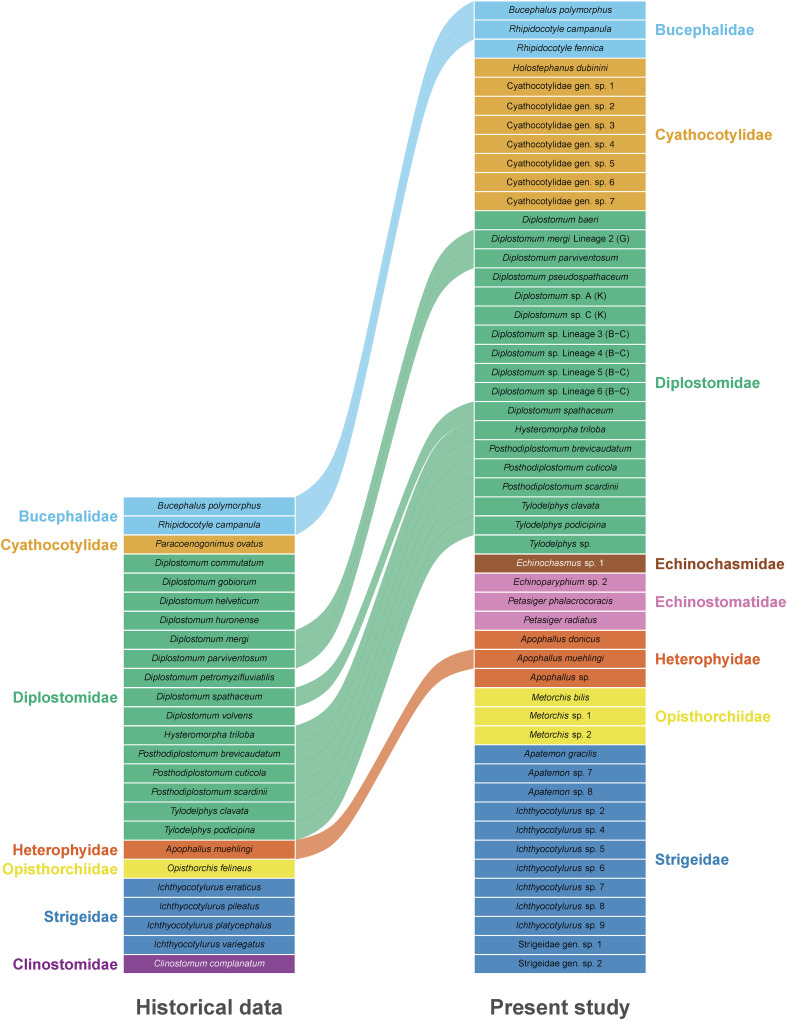
Graphical representation of the historical and present study data of trematode metacercariae parasitising freshwater fish in Lithuania, with curved lines connecting species present in both datasets. Abbreviations: B-C, Blasco-Costa *et al.* [5], G, Georgieva *et al.* [23]; K, Kudlai *et al.* [41].

An analysis of literature records of trematodes reported from freshwater fish in Lithuania showed that metacercariae of 25 species from seven families were recorded prior to our study (Fig. 15). Contrastingly, our study recognised metacercariae of 51 species from eight families. Comparing the species composition, we found similarities at the family level, but notable differences at the species level (Fig. 15). Metacercariae of the family Clinostomidae were previously reported but were not found during the present study. In contrast, metacercariae of the families Echinochasmidae and Echinostomatidae were not previously recorded in freshwater fish in Lithuania but were found in this study. For the remaining families, this study reported a much higher number of species. The Diplostomidae and Strigeidae remained the most diverse families of trematodes in freshwater fish in Lithuania. Comparative analysis of the species diversity in the historical and novel data suggest that at least 12 species were shared between both datasets (Fig. 15). However, it is possible that some of the species from the genera *Diplostomum* and *Ichthyocotylurus* identified only to the genus level in the present study are conspecific to those from historical data, except *Ichthyocotylurus pileatus* (Fig. 13).

Although data on infection rates with metacercariae (occurrence and prevalence) in freshwater fish in Lithuania in the literature are either fragmentary and nonspecific, and therefore cannot be used for analyses, metacercariae of *Ichthyocotylurus* spp. (Strigeidae), *Diplostomum spathaceum*, *Posthodiplostomum scardinii*, *Tylodelphys clavata* (Diplostomidae), and *Paracoenogonimus ovatus* (Cyathocotylidae) were the most numerous in terms of abundance [54, 73].

In addition to changes in species diversity, several changes in the role of different fish as intermediate hosts were observed while analysing historical and novel data. While the Leuciscidae and Percidae remained the primary hosts for the majority of trematode species (Leuciscidae: 20 species in historical data *vs.* 27 in novel data, and Percidae: 15 *vs.* 17 species), the Esocidae, previously the third most important host group (Esocidae: 10 *vs.* 10 species), has been replaced by the Gobiidae (12 species), which is represented by two invasive fish species in Lithuania. Additionally, this study demonstrated that the Gobionidae (3 *vs.* 11 species), Cobitidae (2 *vs.* 7 species), and Tincidae (4 *vs.* 10 species) currently have a higher diversity of metacercariae than previously reported.

### Specificity of metacercariae to their fish hosts

Among the various life cycle stages of trematodes, metacercariae are generally considered to have low host specificity to their hosts. Out of 51 recorded species, host specificity was evaluated for 41 species (Table 2); metacercariae of the remaining 10 species were recovered from one individual of fish host and thus their specificity could not be assessed. Out of the 41 species, 29 (71%) were euryxenous, parasitising fish belonging to at least two families of the same or different orders. Nine species (22%) were stenoxenous, parasitising fish from the same family or genus, while only three species (7%) were oioxenous, parasitising fish of the same species. Metacercariae of *Diplostomum pseudospathaceum*, *Diplostomum spathaceum*, and *Tylodelphys clavata* were the most commonly found species in the widest spectrum of fish hosts, specifically 26 species from 14 families, 26 species from 13 families, and 22 species from 9 families, respectively.

**Table 2:** Distribution of metacercariae among fish host families in the present study (numbers in cells indicate number of infected fish species).

Metacercariae family/species	Fish families																					Total (species/ families)
	Ang	Alo	Ach	Cob	Cyp	Gob	Leu	Nem	Tin	Eso	Lot	Gobi	Odo	Osm	Cot	Gas	Per	Ple	Sal	Ict	Sil	
**Bucephalidae**																						
*Bucephalus polymorphus*			1			1	10					1			1		2					16/6
*Rhipidocotyle campanula*						1	3															4/2
*Rhipidocotyle fennica*					1	1	4		1								1					8/5
**Cyathocotylidae**																						
Cyathocotylidae gen. sp. 1				2								2				1						5/3
Cyathocotylidae gen. sp. 2				2								1										3/2
Cyathocotylidae gen. sp. 3							2					1										3/2
Cyathocotylidae gen. sp. 4							2															2/1
Cyathocotylidae gen. sp. 5			1		1	1	8	1	1	1		1					1					16/8
Cyathocotylidae gen. sp. 7					2		4		1	1							2					10/5
**Diplostomidae**																						
*Diplostomum baeri*							2															2/1
*Diplostomum mergi* Lineage 2 (G)							7															7/1
*Diplostomum parviventosum*						1	1															2/2
*Diplostomum pseudospathaceum*					1	1	10	1	1	1	1	2			1	1	2		2	1	1	26/14
*Diplostomum spathaceum*	1	1			4	2	7				1	2		1		1	3	1		1	1	26/13
*Diplostomum* sp. A (K)					1				1													2/2
*Diplostomum* sp. Lineage 3 (B-C)							6			1					1				1			9/4
*Diplostomum* sp. Lineage 4 (B-C)											1						2					3/2
*Diplostomum* sp. Lineage 5 (B-C)														1					1			2/2
*Diplostomum* sp. Lineage 6 (B-C)																1						1/1
*Hysteromorpha triloba*							7		1													8/2
*Posthodiplostomum brevicaudatum*							3			1							1					5/3
*Posthodiplostomum cuticola*				1	1		10					1										13/4
*Posthodiplostomum scardinii*							4															4/1
*Tylodelphys clavata*					2		10		1	1	1	2					3	1	1			22/9
*Tylodelphys podicipina*							1				1						2					4/3
*Tylodelphys* sp.						1													1			2/2
**Echinostomatidae**																						
*Echinoparyphium* sp. 2				2																		2/1
*Petasiger phalacrocoracis*				1					1													2/2
**Heterophyidae**																						
*Apophallus donicus*										1					1		3					5/3
*Apophallus muehlingi*						1	5															6/2
*Apophallus* sp.				1	2	1	7	1	1	1	1	1							1		1	18/11
**Opisthorchiidae**																						
*Metorchis* sp. 1				2																		2/1
*Metorchis* sp. 2							1		1													2/2
**Strigeidae**																						
*Apatemon gracilis*								1									1					2/2
*Apatemon* sp. 7												1										1/1
*Apatemon* sp. 8												2										2/1
*Ichthyocotylurus* sp. 2																	3					3/1
*Ichthyocotylurus* sp. 4							7										1					8/2
*Ichthyocotylurus* sp. 5						1	1			1							2	1				6/5
*Ichthyocotylurus* sp. 7																	1					1/1
Strigeidae gen. sp. 1					3																	3/1

Abbreviations: 1. Family names: Ang, Anguillidae; Alo, Alosidae; Ach, Acheilognathidae; Cob, Cobitidae; Cyp, Cyprinidae; Gob, Gobionidae; Leu, Leuciscidae; Nem, Nemacheilidae; Tin, Tincidae; Eso, Esocidae; Lot, Lotidae; Gobi, Gobiidae; Odo, Odontobutidae; Osm, Osmeridae; Cot, Cottidae; Gas, Gasterosteidae; Per, Percidae; Ple, Pleuronectidae; Sal, Salmonidae; Ict, Ictaluridae; Sil, Siluridae.

2. Authorities: B-C, Blasco-Costa *et al.* [5], G, Georgieva *et al.* [23]; K, Kudlai *et al.* [40].

### Microhabitat of metacercariae within their fish hosts

Metacercariae were recorded in a variety of tissues and organs of the examined fish, but they were most numerous in muscles, ranging from 1 to > 1 500 specimens per fish. Out of 51 recorded species, 41 were recovered from more than one host individual. Of these, metacercariae of 12 species (29%) were strictly recorded in a single microhabitat within their host, while the remaining metacercariae of 29 species (71%) were found in a range of organs and tissues (Table 1).

In this study, eyes were the most infected organ in terms of species richness and were thus deemed the most important organ for metacercariae in freshwater fish. At least one representative of 7 families reported in the present study were found either inside or on the eye (Table 1). Different parts of the eye – vitreous humour, lens, and retina – were often infected with diplostomid metacercariae from the genera *Diplostomum* and *Tylodelphys*, and a species of *Posthodiplostomum*, *P. brevicaudatum* (von Nordmann, 1832), for which the eye is the preferred microhabitat. Metacercariae of the family Bucephalidae were found inside the eyes or on the eyes (sclera/cornea) in 1.6% of infected fish. Metacercariae of the Cyathocotylidae were found inside the eyes, including the vitreous humour (3 cases), or on the eyes (sclera/cornea) in 4.9% of infected fish. Heterophyid metacercariae from the genus *Apophallus* were found inside the eyes, including the lens (1 case) and vitreous humour (1 case), or on the eyes (sclera/cornea) in 1.4% of infected fish. Strigeid metacercariae, specifically those belonging to the genus *Apatemon*, were commonly found inside the eyes (5% of infected fish), including in the vitreous humour (1 case). A member of the Echinostomatidae, *Petasiger radiatus*, was recorded once in this study and it was found on the eye (sclera/cornea) of its host, *C. gibelio*.

A wide variety of metacercariae (18 species) were found encysted on visceral organs (intestinal wall, liver, kidney, heart, gonads, swim and urinary bladders, *etc.*). Of these, strigeid metacercariae were the most common (26% of infected fish) and most numerous (*I* = up to 600).

Metacercariae of 17 species were found in the muscles of the examined fish. Although members of the family Cyathocotylidae were recorded in different microhabitats within their fish hosts, they were most commonly found in the muscles. Muscle tissue was the preferred microhabitat for diplostomid metacercariae *Hysteromorpha triloba* and *Posthodiplostomum cuticola*. The other metacercariae found in the muscles were bucephalids (3 species), heterophyids (2), opisthorchiids (2), and strigeids (2) (Table 1).

Metacercariae of 14 species from seven families (Bucephalidae, Cyathocotylidae, Diplostomidae, Echinochasmidae, Echinostomatidae, Heterophyidae, and Strigeidae) recorded in this study were found on fish gills (gill arches and filaments). Based on the number of records, bucephalid metacercariae appeared to use gills as the most suitable microhabitat (9.5% of infected fish), but they were also commonly found in the oesophagus.

Metacercariae were often found on the body surface (skin and fins) of examined fish. Various species were enclosed within black capsules, including heterophyids, *Apophallus* spp., and a diplostomid, *P. cuticola*. Metacercariae of *Holostephanus dubinini* Vojtek & Vojtková, 1968 and *M. bilis* were rarely recorded, but when found they were encysted on fins. Several representatives of the Bucephalidae and Cyathocotylidae were found on the skin or fins of infected fish. A total of 11 species were recorded on the body surface of examined fish.

Infections with metacercariae in the brain were rare (2.8% of infected fish). Diplostomid metacercariae, particularly those of *Posthodiplostomum scardinii*, are known to infect the brains of fish, and they were also recorded in this study. Additionally, there were a few cases of cyathocotylid metacercariae, Cyathocotylidae gen. sp. 5 (1 case, *I* = 1), heterophyid *A. donicus* (1 case, *I* = 1), and strigeid *Apatemon* sp. 7 and *Ichthyocotylurus* sp. 2 (3 cases, *I* = 1–2) found in the brain tissue, along with several cases of *Tylodelphys* spp. (7 cases, *I* = 1–12) and Cyathocotylidae gen. sp. 5 (1 case, *I* = 6) found in the cranial cavity.

The kidneys of examined fish were only infected with echinostome metacercariae, specifically *Echinoparyphium* sp. 2.

Compared with adult trematodes, for which the gastrointestinal tract of fish is the most important organ of infection, only metacercariae of the Bucephalidae and Cyathocotylidae were found in the intestinal lumen, albeit seldomly.

## Discussion

In this study, we identified trematode metacercariae of 51 species from freshwater fish in Lithuania, more than twice as many as previously reported (25 species). This high diversity was revealed through the examination of diverse and numerous fish hosts, and by applying an integrative taxonomic approach to species identification. As the family Leuciscidae represents a major proportion of the fish diversity in freshwater ecosystems in Lithuania, it was also found to host the highest diversity of metacercariae. A large proportion of metacercariae from the recorded trematode species (39 species, 76%) are parasites of piscivorous birds, while other hosts consisting of predatory fish, non-piscivorous birds, mammals, or reptiles accounted for the remaining 12 trematode species (24%). Thus, the main contributors to this high diversity of metacercariae in fish are likely piscivorous birds occurring in the region.

### Historical *versus* novel data

Our comparison of historical *versus* novel data on metacercariae revealed that family level diversity was generally similar. However, we did find some interesting differences. One of these differences was the absence of representatives of the family Clinostomidae in fish examined during this study. Metacercariae of *Clinostomum complanatum* (Rudolphi, 1814) were previously reported from the muscles and body cavity of *Sander lucioperca*, *Esox lucius*, and different cypriniforms in Lithuania, occurring in 6.6% of examined fish [69]. In our study, however, not a single clinostomid metacercaria was detected, although *S. lucioperca* (*n* = 15) and *Esox lucius* (*n* = 21), as well as various cypriniform fish, were examined (Fig. 2). This finding is somewhat surprising given that the hosts involved in the life cycle of *Clinostomum complanatum* [definitive hosts – fish-eating birds, specifically herons and cormorants (Ardeidae and Phalacrocoracidae); first intermediate hosts – freshwater snails, such as *Radix auricularia* (Linnaeus), *R. euphratica* (Mousson), *R. ovata* (Draparnaud), and *Lymnaea stagnalis* (Linnaeus); and second intermediate hosts – a broad range of species of freshwater fishes and amphibians [74] are common and widely distributed in Lithuania].

Conversely, we detected species during our study that had not been previously reported from fish in Lithuania, with 2.4% of examined fish found to be infected with metacercariae from the families Echinochasmidae and Echinostomatidae. While reports on *Echinochasmus* spp. and *Petasiger* spp. from their mollusc intermediate hosts [27, 37, 83] and bird definitive hosts [89, 95] are known from Lithuania, these species had never before been found in fish, suggesting that these metacercariae were most probably overlooked in previous parasitological fish surveys.

It is worth noting, however, that our records of a species of *Echinoparyphium* sp. 2 in *Cobitis taenia* and *Sabanejewia baltica* were rather unexpected since the second intermediate hosts of *Echinoparyphium* spp. are typically molluscs [92]. Cercariae of *Echinoparyphium* sp. 2 were previously reported from molluscs *Physella gyrina* in North America [26], later from invasive *Physella acuta* in Iceland [61], and recently from *P. acuta* in North America [79]. In Lithuania, the metacercariae of *Echinoparyphium* sp. 2 were found in the kidney of the fish hosts in relatively large number (*Cobitis taenia*: *P* = 93%, *I* = 1–109; and *Sabanejewia baltica*: *P* = 6 out of 6, *I* = 1–28). These high infection rates may cause kidney failure and lead to mortalities due to an insufficient immune response to the potentially invasive trematode species [53]. Molluscs *Physella acuta* were reported in Lithuania in 2019 [8]. At the time, the restricted distribution and low density of this species suggested their recent introduction. However, during the authors’ samplings in the summer of 2025, *Physella acuta* were found to widely occur in the Curonian Lagoon, as well as in the lakes and rivers of eastern and western Lithuania (unpublished data). Parasitological examinations of *Physella acuta* in Lithuania are therefore necessary to confirm its involvement in the life cycle of *Echinoparyphium* sp. 2 and elucidate its role in parasite transmission within the region.

For the remaining six families, Bucephalidae, Cyathocotylidae, Diplostomidae, Heterophyidae, Opisthorchiidae, and Strigeidae, we report a higher number of species compared to previous studies and one previously unreported genus. Compared with historical data, the family Bucephalidae was represented by the same two species from the same genera, *Bucephalus* and *Rhipidocotyle*, with an additional newly reported species, *Rhipidocotyle fennica*. In contrast to earlier findings, the Heterophyidae was represented by three species of the genus *Apophallus* in the present study. Most similarities between the historical data and the data presented in this study were observed for the family Diplostomidae, with this family being represented by the same genera in both datasets. Out of nine species of *Diplostomum* reported previously and 11 species reported in the present study, only three were found to occur in both datasets. This number could potentially be higher, however, due to difficulties in accurate species identification of metacercariae of *Diplostomum* spp. and the lack of DNA sequences in historical data, a more detailed comparison is impossible. Metacercariae of two species of *Tylodelphys* were previously reported. Both these species, as well as an additional species, *Tylodelphys* sp. from *Pseudorasbora parva* [37], an invasive fish species, and in *Salmo salar*, a species of local fauna, were found during this study.

The most striking differences in species diversity were observed for the families Cyathocotylidae and Strigeidae. A single cyathocotylid species, *Paracoenogonimus ovatus,* previously reported from numerous fish in Lithuania with an occurrence of 0.7–100% [73] was not found in this study. Instead, metacercariae of eight cyathocotylid species representing at least four genera were found. Three out of the eight species, Cyathocotylidae gen. sp. 5–7, were identified to potentially belong *Neogogatea*, a genus that has never been reported in Lithuania nor in Europe as a whole. The identification of metacercariae of these species was based solely on molecular analyses. The finding of these metacercariae in fish from Lithuania was unexpected, since the members of this genus are known to parasitise birds of prey in North America and Asia [1]. *Neogogatea* is a small genus currently consisting of four species. *Neogogatea bubonis* Chandler et Rausch, 1947 ex *Bubo virginianus* (Great Horned Owl) and *N. pandionis* Chandler et Rausch 1948 ex *Pandion haliaetus carolinensis* (Osprey) are known from North America, and *N. rauschi* Zazornova, 1995 ex *Haliaeetus albicilla* (White-tailed Eagle) from East Asia. Another North American species, *Neogogatea kentuckiensis* (Cable, 1935) was experimentally obtained from chicks [30]. Recently, sequences of an unidentified species of *Neogogatea* obtained from hooded merganser *Lophodytes cucullatus* (Anatidae) in North America were published by Achatz *et al.* [1, 2]. Although the specimens were not found in a bird of prey, predominant hosts of *Neogogatea* spp*.*, the authors noted that the specimens obtained from *L. cucullatus* were not fully mature (lacked eggs), but still had characteristic traits of *Neogogatea* (*e.g.* lack of ventral sucker and vitellarium in the form of a horseshoe).

There are 29 bird of prey species known to occur in Lithuania, including those from the genera *Bubo* (*Bubo scandiacus*, *Bubo bubo*), *Pandion* (*Pandion haliaetus*), and *Haliaeetus* (*Haliaeetus pelagicus*, *Haliaeetus albicilla*, and *Hieraaetus pennatus*) [52]. Therefore, metacercariae of Cyathocotylidae gen. sp. 5–7 (possibly *Neogogatea* spp.) found in the present study can potentially use any of these birds as their definitive hosts. However, their first intermediate hosts remain to be discovered in Lithuania. Unexpectedly, this genus was represented by three species, suggesting that the true diversity of cyathocotylid trematodes is vastly underestimated even in well studied Europe. Metacercariae identified as Cyathocotylidae gen. sp. 1–3 all belonged to the same genus and were thought to belong to the genus *Cyathocotyle* based on the molecular identification with sequence accession number MF124273 used as reference. However, a recent sequence of *Cyathocotyle bushiensis* Khan 1962 obtained from an adult specimen was included in the phylogenetic analyses by Achatz *et al.* [2], demonstrating its distant position from the sequences identified as *Cyathocotyle prussica* Mühling, 1896 and *Cyathocotyle* sp. (GenBank accessions MN726941 and MN726942).

Within historical records, metacercariae of the Strigeidae are represented by four species from a single genus *Ichthyocotylurus*, all of which were not linked to the novel data since none of the detected species could be identified to the species level. Instead, our study recognised seven species of *Ichthyocotylurus*, with one found in percid fish clustering with *Ichthyocotylurus* sp. 2 reported from *Perca flavescens* in North America [50]. Metacercariae of this species, together with those of *Ichthyocotylurus* sp. 4 and *Ichthyocotylurus* sp. 5, were found more often and in a wider spectrum of fish hosts. The remaining four species were found in one species of fish host from various families. Out of three species of *Apatemon*, only metacercariae of *A. gracilis* were identified to the species level. The remaining two unidentified *Apatemon* species were found exclusively in invasive fish species, *Neogobius fluviatilis* and *Neogobius melanostomus* [43]. Two other members of the Strigeidae found in the present study most likely belong to two genera. One of them was found in cyprinid fishes, *Carassius carassius*, *Carassius gibelio*, and *Cyprinus carpio*, while the other was found in a single leuciscid species, *Leucaspius delineatus*.

Given the shortfalls of morphology-based identifications, reports of underrepresented and unrecognised diversity, specifically among digenetic trematodes, have become common in studies applying molecular methods for species delineation and identification [5, 18, 42, 63, 82]. It was therefore expected that the diversity of trematode metacercariae in fish in Lithuania would be somewhat higher, with at least a few previously undetected species being discovered in the present study. However, an increase in diversity of more than 100% compared to historical records far exceeded our expectations and we accordingly offer several explanations.

First, a considerable number of species may have been overlooked in previous studies since the identification of metacercariae was solely performed using morphological identification techniques. Second, more thorough examinations of fish in the present study contributed to a high number of metacercariae found. For example, *Petasiger radiatus* and *Petasiger phalacrocoracis* (Echinostomatidae), which were detected in this study, were both reported in Lithuania in their mollusc and bird hosts, although they have never been detected in fish in the region. However, in line with this logic, the presence of *Echinoparyphium* sp. 2 (Echinostomatidae) provides a somewhat confounding argument here, which we postulate might be due either to the recent arrival of this species with its invasive mollusc host, or to this species finding its suitable intermediate mollusc host upon arrival with another host (possibly birds). Third, the appearance and proliferation of invasive fish hosts and their participation in transmission pathways of trematodes as the second intermediate hosts likely contributed to an increase of trematode species diversity. Invasive fish species have possibly facilitated the establishment of metacercariae of several species. For example, two species of the genus *Apatemon* were found exclusively in invasive fish, *Neogobius fluviatilis* and *Neogobius melanostomus* [43], and no evidence was observed for these species being shared amongst native and introduced freshwater fishes in Lithuania. *Neogobius fluviatilis* and *N*. *melanostomus* seem to be the most successful fish invaders in Lithuania.

In addition to the changes in species diversity, our study revealed notable differences in the role of these invasive fish species in transmitting metacercariae. Among the analysed fish in the historical data, the Leuciscidae, Percidae, and Esocidae were the main hosts involved in the transmission of metacercariae in Lithuanian freshwaters. However, our novel data showed that pike (Esocidae) were replaced by invasive gobiids. Although it is impossible to compare the infection rates between historical and novel data, it seems that the invasion of new fish hosts had a positive impact on local trematode transmission, which was similarly reported by Hohenadler *et al.* [31]. We postulate that this is potentially due to parasite spillback, whereby non-native species become important for circulating local parasite species and keeping them persistent [34, 53].

### Host specificity

Metacercariae are generally considered to have low host specificity [62]. However, this hypothesis has been difficult to evaluate due to challenges in identifying metacercariae to the species level based on morphological analysis alone. In this study, where identification of metacercariae was based on integrated morphological and molecular analyses, we confirm that the host specificity of metacercariae to fish hosts was low, with most species being euryxenous (71%), being found in fish belonging to different families of the same or different orders. Those that were reported from a single family, genus, or species of fish were found rarely and, for some, host specificity needs to be further assessed.

Correspondingly, our results do not conform to the statement of Blasco-Costa and Locke [6] implying that diplostomid metacercariae exhibit higher host specificity than previously thought. Out of the 18 diplostomid species found in the present study, only five [*Diplostomum baeri* Dubois, 1937, *Diplostomum mergi* Lineage 2 of Georgieva *et al.* [23], *Diplostomum* sp. C of Kudlai *et al.* [41], *Diplostomum* sp. Lineage 6 of Blasco-Costa *et al.* [5], and *Posthodiplostomum scardinii*] were reported from fish belonging to the same family. However, when comparing the host spectrum of these five species available in the literature (confirmed with molecular methods [81, 87]), there were only three species reported to parasitise fish of the same family, namely *Diplostomum* sp. C of Kudlai *et al.* [41] found in lenses of only leuciscids, *Diplostomum* sp. Lineage 6 of Blasco-Costa *et al.* [5] found in lenses and retina of gasterosteids, and *Posthodiplostomum scardinii* found in the brain of leuciscids.

Locke *et al.* [50, 51] concluded that host specificity in lens-infecting *Diplostomum* spp. is lower compared to those infecting non-lens tissues. Seven out of the 11 species of *Diplostomum* recorded in this study were lens-infecting and showed low host specificity; they were found in fish belonging to different families. One species, found exclusively in the retina, also showed low host specificity as it was found in osmerid and salmonid fishes. Out of the three remaining species of *Diplostomum* isolated from the vitreous humour and lens, the retina and lens, or the vitreous humour, retina, and lens, only *Diplostomum* sp. Lineage 6 of Blasco-Costa *et al.* [5] was found in fish belonging to a single family, the Gasterosteidae.

When comparing the results of the present study to those studies where metacercariae of *Posthodiplostomum* were identified using molecular data, our results align with the results of Pérez-Ponce de León *et al.* [64] who found *Posthodiplostomum* spp. in multiple families of fish. In our study, metacercariae of *Posthodiplostomum brevicaudatum* were found in four fish species belonging to three families (Esocidae, Leuciscidae, and Percidae) from three orders, while metacercariae of *P. cuticola* were found in 13 fish species belonging to four families (Cobitidae, Cyprinidae, Gobiidae, and Leuciscidae) from two orders.

Sándor *et al.* [76], in their study on heterophyid metacercariae from freshwater fish in Hungary applying molecular identification, reported that metacercariae of *Apophallus* are specific to the family of fish. Accordingly, the authors reported that metacercariae of *Apophallus muehlingi* and *Apophallus* sp. infect cyprinid fishes, whereas those of *Apophallus donicus* infect percid fishes. It must be noted, however, that the authors only examined cyprinid and percid fishes. The results of our study contradict the results obtained by Sándor *et al.* [76] in that *Apophallus muehlingi* was found in fish from two families, *Apophallus donicus* was found in fish from three families, and *Apophallus* sp. was found in fish from ten families, thereby suggesting that *Apophallus* spp. do not in fact demonstrate host specificity to fish family. To settle the debate of host specificity, future studies conducted in different regions should investigate a wide variety of fish species and apply integrated methods to the species identification of metacercariae.

### Microhabitat specificity

Cercariae penetrating the body of fish may become encysted either at the place of penetration or may migrate through the fish body to a specific microhabitat that would be suitable for their survival and transmission efficiency [25]. Compared to adult trematodes that tend to occupy different parts of a single organ of fish – the digestive tract – metacercariae occur in different organs and tissues of freshwater fish, including the heart, liver, kidney, eyes, and brain, and often in high densities for extended periods of time, thereby explaining their high pathogenicity. Transmission of metacercariae to their definitive hosts typically requires that the fish host be debilitated or otherwise vulnerable to predation. Metacercariae are therefore environmentally, economically, and socially important as they can reduce fish production, affect fish health and growth, make fish more vulnerable to other infections, and even result in mass fish mortality. Although the pathogenic effect of metacercarial infection in fish was not assessed in this study, when considering the high prevalence (72%) and number of multiple infections (55%), it is possible that metacercariae are key players in ecosystem dynamics and curators (and indicators) of fish and ecosystem health. Among metacercariae found in freshwater fish in Lithuania, species belonging to the families Echinochasmidae, Heterophyidae, and Opisthorchiidae potentially pose a risk for human infection, especially if freshwater fish are consumed undercooked or raw [16].

In this study, the eyes and muscles harboured the highest number of species and density of metacercariae, respectively. Metacercariae demonstrated different levels of habitat selection, with some being habitat-specific and others being rather opportunistic. Similar to previous reports [5, 59, 73, 87], members of the Diplostomidae demonstrated complex microhabitat selection within their fish host. For example, compared to strigeid metacercariae that were mainly found encysted on different internal organs of fish, diplostomid metacercariae parasitise different organs and tissues of fish. While metacercariae of *Diplostomum* spp. and *Tylodelphys* spp. infected different parts of the eye or brain, metacercariae of each out of three species of *Posthodiplostomum* were found in a unique microhabitat not overlapping with other *Posthodiplostomum* spp.: metacercariae of *Posthodiplostomum brevicaudatum* occurred in the eyes, *Posthodiplostomum scardinii* occurred in the brain, and those of *Posthodiplostomum cuticola* were found on the body surface, gills, muscles, and even on the intestinal wall of invasive *Neogobius fluviatilis*. Metacercariae of the Heterophyidae were consistently found in the same microhabitats within their fish host, which were skin, fins, gills, eyes, and muscles. Metacercariae of the remaining families showed little microhabitat selection; however, metacercariae of the Cyathocotylidae, which were found in different microhabitats, tended to show a preference for fish muscle tissue.

While microhabitat preferences are well-documented in individual systems (*Diplostomum* spp. in fish [4, 5, 51], *Ribeiroia* spp. in amphibian limb buds [9, 33]), systematic comparative analysis across species of metacercariae in fish hosts is lacking. Understanding the use of microhabitats by trematode metacercariae allows a deeper understanding of both the ecological interactions between different species within the host (for example, how metacercariae impact fish behaviour, competition, or survival) and evolutionary interactions between hosts and their parasite (for example, how fish develop defence mechanisms and trematodes develop strategies to overcome them). Additionally, intraspecific interaction between parasites in the same microhabitat remains a long-standing question in parasitology, with several studies performed on metacercariae in fish. For example, Kennedy [36] and Désilets *et al.* [15] reported on negative associations between *Diplostomum pungiti* Shigin, 1965 (syn. *Diplostomum gasterostei* Williams, 1966) and *Tylodelphys* spp. in the vitreous humour and between *Diplostomum* spp. in lens, respectively.

Future studies should therefore follow a similar approach to the present one, and in particular consider the unique microhabitats in which the trematode species occur and examine a large range of fish hosts sampled from a diverse array of heterogeneous freshwater habitats at large spatial scales. This will help to build a better and more complete picture of the host and microhabitat specificities of trematode parasites, potentially unravelling their elusive interactions within their hosts and natural systems.

## Conclusion

Our study revealed a much higher diversity of trematode metacercariae in freshwater fish in Lithuania compared to historical morphology-based surveys from the region. This finding aligns with recent studies applying molecular identification techniques, which have consistently revealed unrecognised diversity across different trematode taxa. However, most genetically recognised trematodes, specifically at the larval stages, remain unidentified as they were not linked to their adults; through comparative analysis of DNA sequence data, we were only able to identify 21 species to the species level in the present study (~41%). Nevertheless, gathering molecular data from correctly identified adults will prove pivotal to identify those species that we could currently only identify to the genus or family levels.

The application of molecular identification techniques has revealed the presence of trematode species in European freshwater ecosystems that were previously known only from other continents, yet they remain unidentified to species level. In the present study, these were *Echinoparyphium* sp. 2 and *Ichthyocotylurus* sp. 2, previously reported in molluscs and fish in North America. This taxonomic uncertainty raises questions about whether their occurrence represents broader geographical distributions than previously known or represents recent introductions. Additionally, the metacercariae of Cyathocotylidae gen. sp. 5–7 (possibly *Neogogatea* spp.), a genus never before reported in Europe, requires further species identification and confirmation.

The rapid growth of molecular trematode studies has created a bias where species are often attributed to the region where they are first genetically characterised, even though they may have much wider natural distributions facilitated by diverse and poorly understood host spectrums or transmission pathways. Consequently, while these studies provide valuable baseline data for trematode larvae, the lack of knowledge about trematode adults limits our ability to elucidate their life cycles and accurately assess invasion risks. With ongoing and continuous global environmental change, as well as the proliferation of non-native species, the spread and homogenisation of trematode diversity amongst fish, which lack evolutionarily adaptative defence mechanisms is extremely worrying, specifically if this gives other more deleterious non-native species an added competitive advantage over local fauna.

## Data Availability

All data relating to the present study are embedded within this manuscript and Tables S1–S2. Any additional data are available from the authors upon reasonable request.

## Supplementary material

*Table S1*: Water bodies, localities and coordinates of sampling sites. *Table S2*: Summary data for the metacercarial isolates used for the generation of the *cox*1, *nad*1, 28S, and ITS2 sequences in the present study. The Supplementary material of this article is available at https://www.parasite-journal.org/10.1051/parasite/2025067/olm.
