# Supplementary material for: Comparison of historic and novel data reveals higher contemporary diversity of trematode metacercariae in freshwater fish
Source: Parasite. 2026 Jan 6;33:1. doi: 10.1051/parasite/2025067 (PMC12779263; doi:10.1051/parasite/2025067)
Supplement: Supplementary file 1 — Table S1: Water bodies, localities and coordinates of sampling sites. [file parasite-33-1-s1.pdf]

Comparison of historic and novel data reveals higher contemporary diversity of trematode metacercariae in freshwater fish

Olena Kudlai, Rasa Binkienė, Vytautas Rakauskas, Nathan Jay Baker

Table S1. Water bodies, localities, and coordinates of sampling sites. Coordinates are in WGS84 (North, East).

No.	Water body	Locality (nearest village or city)	Coordinates
1	Curonian Lagoon	Ventė	55.346735, 21.175857
		Kiaulės nugaros sala	55.656278, 21.135288
		Klaipėda	55.718409, 21.102401
2	Kaunas reservoir	Arlaviškės	54.808204, 24.173606
		Grabuciškės	54.89238, 24.146082
		Kapitoniškės	54.84112, 24.201536
		Kruonis	54.816351, 24.23471
		Lašiniai	54.817328, 24.233604
		Vaiguva	54.801605, 24.231826
3	Nemunas River	Leitė	55.275627, 21.414112
		near Leitė	55.275165, 21.414807
		Lipliūnai	53.987219, 23.914091
		Sudargas	55.051171, 22.643179
		Vilkija	55.029198, 23.58906
4	Neris River	Mykoliškiai	55.022778, 24.115616
		Paliepė	54.863363, 25.745182
		Saidė	54.721319, 25.045303
		Skirgiškės	54.837704, 25.375939
5	Žeimena River	Kaltanėnai	55.252704, 25.990691
		Pažeimenė	55.028723, 25.847220
		Šulikiškiai	55.028715, 25.847322
6	Jūra River	Mociškiai	55.107601, 22.173838
7	Upė River	Paupys	55.320702, 22.894767
8	Merkys River	Trasninkas	54.107813, 24.276172
9	Venta River	Kuodžiai	56.378274, 22.225903
10	Širvinta River	Stolaukis	54.548675, 22.943493
11	Pilvė River	Antanavas	54.713178, 23.319018
12	Grūda River	Puvočiai	54.119233, 24.304610
13	Drūkšiai Lake	Raipolė	55.649338, 26.583776
14	Dūsia Lake	Metelytė	54.328986, 23.671156
15	Maušelis Lake	Statiškė	54.26975, 23.828778
16	Gabris Lake	Laukesa	55.763536, 26.276487
17	Bedugnis Lake	Vievis	54.783468, 24.812681
18	Šėlinis Lake	Kochanovka	55.173501, 26.258422
19	Privalskis Lake	Aukštadvaris	54.573396, 24.524770
20	Lake in Statiškės forest	Statiškė	54.278166, 23.822533
21	Dysnai Lake	Varnos	55.493525, 26.368139
22	Swamp	Dvarviečiai	56.110158, 21.824709
23	Pond	Alytus	54.400521, 24.009722
24	Gravel pit	Klaipėda	55.603325, 21.200070
